# Sculpting the Glauberg “prince”. A traceological research of the Celtic sculpture and related fragments from the Glauberg (Hesse, Germany)

**DOI:** 10.1371/journal.pone.0271353

**Published:** 2022-08-11

**Authors:** Martin Trefný, Doris Mischka, Michal Cihla, Axel. G. Posluschny, František R. Václavík, Wolfram Ney, Carsten Mischka

**Affiliations:** 1 Institute for Pre- and Protohistory, Friedrich-Alexander-Universität Erlangen-Nürnberg, Erlangen, Germany; 2 Institute of History, University of J. E. Purkyně, Ústí nad Labem, Czech Republic; 3 Department of Monument Diagnostics and Conservation, Institute of Theoretical and Applied Mechanics, Prague, Czech Republic; 4 Keltenwelt am Glauberg, Glauburg, Germany; 5 Institute of Historical Sciences, University of Pardubice, Pardubice, Czech Republic; 6 Institut für Altertumswissenschaften, Johannes Gutenberg Universität Mainz, Mainz, Germany; University at Buffalo - The State University of New York, UNITED STATES

## Abstract

Article presents the results of a complex traceological research of the famous statue of the „prince”of Glauberg, found in an Early La Tène funeral complex in Glauberg (Hesse). Research focused also on two other fragments of related sandstone sculptures, found together with the Glauberger prince. The sandstone „prince”of Glauberg was already in the past a subject of many archaeological studies. Nevertheless, all or absolute majority of them were focused on aspects of art historian nature or on the question of the origin, role and function of such sculptures in the Early Iron Age Central Europe. On the contrary, the aim of our research is oriented exclusively on the questions related to the manufacture of this sculpture, identification of used sculptor´s tools and applied working techniques. Our research was realised by means of digital documentation followed by the aplication of traceological methods. The character of the survived working traces on the sculpture´s surfaces was studied by mechanoscopy, while the material of used tools was determined by X-ray fluorescence. The reconstructions of used tools were compared with the existing tools as represented by the Iron Age archaeological finds. This comparison was oriented on the most relevant regions of developed La Tène culture, particularly on South Western Germany and Bohemia. However, also other relevant area, significant as the possible source of inspiration of Celtic sculptors for the creation of the monumental sculpture–Apennine peninsula, was taken into consideration. Our research revealed individual steps and phases during the sculpture´s manufacture, enabled the reconstrucion of used tools and confirmed real existence of such tools in mentioned regions. Finally it has brought first indices of the necessity of the distinguishing between ideological and technological aspects of related Celtic sculpture, when considering possible influence of Apennine peninsula on transalpine Central Europe.

## 1. Introduction

Iron Age statues, stelae and anthromorphic pillars in Central Europe have been produced during the period from the 7th to the 3rd century BC mainly in Baden-Wurttemberg, Rhineland-Palatinate, Hesse and Eastern France and were of particular significance in funerary cult and representation [1]. Similar function is considered also for the Pre-Roman sculpture in Southern France [2–4] or in Iberian peninsula [5–7].

The use of the sculptures in Central Europe as prestigious or commemorative objects near burials during the Early Iron Age is considered to be derived from the Italic culture of Picenum. The initial phase of the utilization of the monumental scupture is in Central Europe connected with the end of the 7^th^ and beginning of the 6^th^ century BC and first such sculpture has emerged in Baden-Württemberg in Southwestern Germany (type Stockach) [1]. This type, dated to the transitional phase Ha C2/D1 represented simple and schematic representation of a human body, expressed by three main parts–a head, neck and body complemented by the incised horizontal zig-zag line. It also seems that the use of the stone monumental sculpture has been during this initial phase restricted only to small territory in central part of Baden-Württemberg. It is indicated by the fact that all seven sculptures belonging to the type Stockach (Birkach, Rottenburg am Neckar, Stammheim, Tübingen-Kilchberg) were found in a confined area along the river Neckar in the vicinity of Tübingen [8].

The well-known sculpture of a warrior from the tumulus in Hirschlanden dates back to ca 500 BC and represent a new approach in comprehension of the human figure compared to earlier schematic works [9–12]. The Hirschlanden hero is rendered as a full figure with depiction of all relevant part of the body, including the belt, dagger, perhaps mask on the face and a hat. Nevertheless, it shows two different levels of the rendering. The thigs and calf are much closer to real relevant parts of the human body and relatively similar to the feet of Greek kouroi, as well as to the feet of the prince of Glauberg- a subject of this paper, while the upper part of the body is flat, non-plastic and clearly distinguished from the lower half of the figure.

The progress in the sculpture made in broader Central Europe around 500 BC or slightly later is demonstrated by the figures from Vix-Les Herbues [13–15]. Fragments of two sculptures of sitting persons were found near the entrance of the area limited by the rectangular ditch. These statues are significant not only as an evidence of capability of the rendering of the sitting figure, but also due to the depicted jewellery or weapons. The torc of the first sitting person is similar to the real piece of the jewellry made of gold and found in the famous grave of the princess from Vix. The shield with central vertical rib-the weapon held by the second sculpture-is perhaps the earliest representation of this type of the shield [14].

Leaving aside the prince of Glauberg (see ultra), another important piece of sculpture is represented by the upper part of the head wit the mistletoe cap found in Heidelberg and dated to the period of 5^th^-4^th^ century BC [16]. Although major parts of the head were not preserved, the surviving elements indicate clear relationship with the relevant parts of the prince from Glauberg.

Another significant Central European find is the pillar from Pfalzfeld dated to the 5^th^–early 4^th^ century BC [17–19]. However, the Pfalzfeld pillar is not an example of an antropomorphic sculpture in the right sense, since its form corresponds to rectangular conical stele, with globular lower part. Nevertheless, the lateral decoration in the form of the human heads with the mistletoe cap and relevant floral motives fully coresponds with the similar depictions of the Early La Tène art.

The group of the pieces belonging to the type Pfalzfeld includes also the stele from Steinenbronn, dated already to the phase LT B–rectangular block on which only a little of figural decoration was preserved [18, 20] or the stele from Holzerlingen [1, 16, 20]–a quadratic monument with schematically rendered arms and a head with mistletoe cap, dated back to the period of 5^th^-4^th^ century BC.

Some stelae of the later period could have served as a marking of the flat graves. This is the case of so called anikonic monuments, without any depiction, for example from Enkenbach-Alsenborn, dated back to the phase LT C [21]. Similar purpose could have had aniconic stelae from Rheinland-Pfalz, from Hassloch, Bad Dürkheim or Schifferstadt [1, 21, 22].

One of the latest antrophomorphic monuments from the Iron age in the German territory are two wooden animal figures from Fellbach-Schmiden [23, 24], bearing on their surfaces human hands. This indicates that both animals were originally parts of greater group including a human figure. They could have been dated by means of dendrochronology to 127 BC. Two stone heads from Freinsheim and Arzheim could have been made in the same period.

An integral part of Central European Iron age culture is also the Bohemian bassin. However, this territory yielded up to now only two stone sculptures. The most known is the stone head from Mšecké Žehrovice in Central Bohemia [25, 26]. The marlstone head bears typical attributes of the La Tène art, including th great torc, typical hair-dress, beard and other features which enable to date it to the 3rd century BC. On the contrary, the sandstone head from oppidum Závist near Prague was originally connected with Early La Tene settlement of this site [27], although there were also such opinions that have refused its authenticity [28–31]. Recent analysis revealed that this head was produced usinng the manufacturing stonemason´s methods well known in Antiquity. Moreover, particular stylistic traits and other aspects could indicate rather later period than La Tène [32].

The archaeological contexts in Central Europe with occurrence of the monumental stone sculpture are predominantly represented by the sanctuaries and cemeteries. It is necessary to stress that the finding context is a crucial aspect for the interpretation of such sculpture. On the other hand many of the finds of such sculpture have unknown finding context, so the similar considerations have sometimes only a speculative value.

The most sculptures with known finding context were found in the vicinity of the burial mounds. This is the case of finds from Tübingen-Kilchberg, Rottenburg, Stockach, Hirschlanden and Glauberg. The sculptures were found near the perimeter wall of the tumuli (Tübingen-Kilchberg, Hirschlanden), as a part of the stone covering on the grave in the tumulus (Tübingen-Kilchberg, Rottenburg am Neckar), separately inside the tumulus (Stockach) or in greater distance (tenths or hundreds of meters) from tumulus (Glauberg, Hirzenhein, Stammheim). All these contexts allow considerations on the original placement of the sculptures on the top of the tumuli, beside them or in a shorter distance from them. The function of the sculptures in these cases might have oscilated between the representation of burried persons (Hirschlanden, Glauberg), marking of the tumulus as a burial of the member of the social elite or a subject of the cult of the burried person or ancestral cult etc [8]. However, it is true that when the sculptures were almost never found in the place where they originally stood, their interpretation will be always speculative [21].

The situations where it would be possible to consider the interpretation of the sculpture as a part of a sanctuary are less frequent. Taking into consideration the sculpture in Central Europe, such role may be supposed only in a few cases. First one is the rectangular precinct in Vix-Les Herbues. Both sitting figures were found here near the entrance probably in or near their original place. This rectangular structure has been probably a sanctuary, but possibly connected with a cult of the dead, since one of the sitting persons is interpreted as a depiction of a person burried in a famous female grave in Vix [33].

The function of a sanctuary cannot be excluded also in case of the Viereckschanze from Fellbach-Schmiden, where three wooden animal figures were found in a well located in the interrior of precinct [23, 24]. However, the character of a local cult seems unknown.

Although, the function of the sculpture in sanctuaries may be connected with a representation of a particular person, as wee have seen in case of one of the figures from Vix-Les Herbues, they may have also other functions. Here the most logical interpretation may be the personifications of deities. One of the attribute of the deity may be, after some scholars, so caled mistletoe cap [17, 34, 35]. It is true, that no sculpture from here mentioned sanctuaries wears this attribute as well as such, here mentioned, figures with this cap cannot be simply interpreted as scultprues from sanctuaries. Nevertheless, there exist one example, where the mistletoe cap and a cultic precinct as a place of finding of such sculpture may be linked together. It is the acropolis of the Early La Tène hillfort of Závist near Prague, where a stone fragment most likely from a mistletoe cap has been uncovered [27]. Although the possibility of the function of the sculpture as a representation of the deities may be accentuated by the mentioned connection, it is obvious that the sculpture may have also many other functions and roles [8].

It is striking that no one of the mentioned examples of the stone sculpture of Iron Age from Central Europe has been found in a settlement. The situation is different in other significant zone with the emergence of the Pre-Roman stone sculpture–in Southern France. Also here the utilization of the monumental stone sculpture starts in the timespan of 7th-6th century BC, predominantly with the depictions resembling the busts, as attested by the sculptures from Beaucaire [3, 17], Castelnau-le Lez [3, 36–38], Corconne [3, 39, 40] or Saint Bonnet du Gard [3, 38]. However, also the later period of 6th-5th century BC knows the representations in a form of busts, as for example pieces from Sainte-Anastasie [3, 36, 38, 40, 41], although these seem to be in many aspect more progressive as the abovementioned ones.

The overall progress of a sculpting of the human figure in the period of 6th-5th century BC is well documented by the „sitting”sculptures from Roquepertuse [2, 3, 41, 42]. The variability of the sculptures of this period is documented also by the figures of which only a torso survived, as is the case of finds from Lançon-de-Provence [3, 43, 44], Lattara [3, 45, 46], Nîmes [3, 41, 47, 48] or Castelnau-en-Lez [40, 49], where only a head has survived. Some finds document also for this period or more precisely for the late 6th century BC also the use of the wood as a material for the sculptures, as seen in case of the find from Securre, Saône [29, 50–52]. The evolution of the stone sculpture in Southern France continues to the later phases until the Gallo-Roman period, as well as in Germany to the Late La Tène period. Nevertheless, the density of the sculpture here is much higher than in the regions of Central Europe [8].

As for the findig contexts of the scultpure in Southern France, the finds from the period of the greatest significance for out topic–an Early Iron Age–or in terms of absolute chronology from the 7th-5th centruy BC may be found frequently in the settlements. This is for example the case of finds from Beaucaire, Castelnau-le Lez, Saint Bonnet du Gard or Lattara. Questionable is the interpretation of the context of „sitting”figures from Roquepertuse, that has been originally interpreted as a sanctuary but later also the considerations in the terms of a public place located in a settlement emerged [53]. The comparison of the Early Iron Age finding context of the sculptures in South France and Central Europe, where it was found mainly in connection with the funeral monuments, indicate the different perception and significance of the stone sculpture in both regions.

Another significant zone with the emergence of the Pre-Roman stone sculpture is the peninsula of Istria in the Northern Adriatic zone. Here the two sculptures of Nesactium may be mentioned [1, 16]. In case of the first sculpture only upper parts of thigs and lower part of the belly including erected fallus were preserved. It is most likely that this sculpture has originally represented a standing warrior, similarly as Hirschlanden or Glauberg heroes. From the second sculpture only a part of the chest with crossed arms in typical gesture has been preserved. Both sculptures are to due to their chronology (second half of the 6th century BC) and formal characteristics considered to be the possible link between the Italic sculpture and abovementioned standing figures of Central Europe [1, 8, 16].

The last group of the Pre-Roman scupture, and probably most important for the evolution of related Central European counterparts, is the sculpture of central Apennine peninsula. The earliest pieces here may be compared with the schematic works of the group Stockach in Southern Germany. It is the case of the stele from Guardiagrele [48, 54, 55] showing the flat schematic design and simplified details of the face as well as necklace or the kardiofylax or shield on the belt, close to the type Capena. Similarly as some pieces of the Stockach group, also the stele from Guardiagrele is dated back to the 7th century BC [56–58].

Special position in the group of the Italic sculpture has so called warrior of Capestrano [55, 59, 60], dated back to the mid-6th century BC. Some elements as sharply profiled shins and calfs, similar gesture of the arms, standing position, depiction of the weapons that are typical for the region, and others indicate that this sculpture -the representation of local king–could have most likely served as an inspiration for the sculptors of Hirschlanden and Glauberg warriors [8, 61], than the Greek kouroi, as originally considered [9, 62]. In connection with Capestrano warrior, a fragment from Collelongo, dated back to the 6th century BC may be mentioned [63]. Unfortunately only the thigs and shines were preserved from the figure of standing warrior. Nevertheless, it is obvious, that the calfs and especially sharp shines are profiled in the same way as in case of Capestrano figure.

Also other Italic sculptures are relevant to the standing figures of warriors from Hirschlanden and Glauberg. For example a female torso from Capestrano [55] or standing figure from Casale Marrittimo [64], dated even to the mid-7th century BC [55] show similar gesture of the folded arms on the chest. An early date seems here a bit surprising, but this sculpture is not in the period of 7th century absolutely isolated phenomenon, since also the well known stone head from Numana with the helmet close to the type Novilara dates back to the end of the 7th century [55]. Comparable, but not completely the same gesture as abovementioned pieces shows also the fragment of the torso and arms from Atessa [63] or from Rapino [63], both dated to the 6th century BC [63].

Many of the stelae and figures from the mentioned European regions show one common element-the typical gesture of crossed or folded arms on the belly or chest. It is also the case of most relevant full standing figures, such as kings or warriors from Capestrano [56, 63, 65], Hirschlanden [9–12] or Glauberg, where it may be assumed that the statue represents a historic personage or its role and status within the society, since a burial with exactly the same objects as shown on the statue were found in the tumulus next to the statue [48, 66].

This specific gesture of the arms also occurs on other examples in the area north or northwest of the Alps. The position of the arms on the sandstone statue from Mont-Saint-Vincent (Saône-et-Loire) is somewhat unclear. Despite that, it is visible that at least one hand is laid on the breast [8, 29]. The gesture of the statue from Rai-Breitenbach, Breuberg (Odenwaldkreis) is much clearer and the positions of both arms resting on the breast can be clearly distinguished [1, 8, 48, 67]. Less distinct is the position of the arms in the case of the statues from Rottenburg am Neckar, Kr. Tübingen [1, 8, 68] or Stammheim, Kr. Calw [1, 8, 69]. The basic problem of the three last mentioned stelae is the unclear or imprecise chronology [18]. The statue from Mont-Saint Vincent comes from an unknown context and is dated, based on the stylistic analysis, to the period of the 5th or 4th century BC, the statue from Rai-Breitennbach to the 6th-5th century BC, the stele from Rottenburg am Neckar to the 7th century BC (but possibly also the Final Neolithic) [70], and the stela from Stammheim is assigned to the 6th century BC.

However, searching for the depiction of the mentioned gesture on the stone stelae or pillars, we have to shift our attention to other regions outside southwestern Germany and also to much earlier periods. In his publication on the Lumbrein stele, U. Schwegler [71] writes about ca 500 examples of stelae, pillars or menhirs distributed between the Pyrenees and the Black Sea. He has distinguished seven groups, many of which include stelae with the arms positioned in the described gesture [71]. Schwegler’s group 7 includes the statues from Glauberg, Hirschlanden or Capestrano and other pieces with the same or similar gesture. These stelae occur mainly in Central Germany, Central and Eastern France, Switzerland, Central and North-Central Italy, Greece, East Romania and Central Eastern Ukraine [71]. The spatial non-homogeneity of this distribution reflects also the different chronology of these stelae. The finds from Ukraine and Greece are Late Neolithic, the stela from Sietschen-Lumbrein falls into the period from the Aeneolithic until the Iron Age, the Italian, French and German finds belong to the Iron Age [72, 73]. The research of T. G. Schattner registers nearly 40 examples of stelae, statues and statuettes with the same specific hand gesture, spreading from Iberia to what is now Northeastern Turkey [74].

The affinities in certain details, as well as in the overall appearance of the sculptures from the north as well as south of the Alps, and in some cases also from the East Mediterranean Greek milieu, indicate the possibility of mutual cultural exchange between these regions, demonstrated in the monumental stone sculpture. Such assumptions need not be absolutely illogical, because the regions in Central Europe and the area of the Apennine peninsula show many signs of reciprocal interactions during the Early Iron Age [75–89]. In the 6th and 5th century BC the circum-alpine region opens up to the Mediterranean world and culture. During this period prestigious objects were imported from the south, such as Greek or Etruscan bronze vessels or Attic pottery. Apart from direct imports of particular items, the import of ideas (i.e. import of the aspects of immovable character, such as various elements in the stone architecture or the use of scultpures in a funeral context) should also be mentioned. For example, below the Mont Lassois hillfort, two statues have been found in what is interpreted as the “sanctuary of Vix”. One of these might have represented the famous “princess of Vix”, a very rich burial of a late Hallstatt woman with such luxurious grave goods as a decorated golden torque and a huge bronze crater, made in Greek Southern Italy.

Research on the transalpine Iron Age sculptures resulted in many studies [4, 21, 29, 48, 72, 88–93].

However, these studies mainly focussed on the social significance of these monuments or their role in funeral rites connected with the contemporary elite, and related aspects. The main scientific approach in these studies comprised stylistic analysis and comparison with their counterparts in the Italic or Greek millieu. The present article, on the contrary, aims to research one of the most famous sculptures–the Glauberg statue and related fragments–exclusively from the point of view of technological analyses, which allow the reconstruction of the manufacturing processes, the tools used, and other aspects of technical nature.

As regards similar research in the past, only a few examinations of tool marks or traces were realised by J. Röder [94] on the statue from Hirschlanden, the earliest full plastic statue north of the Alps. He noticed that several kinds of pointed and flat tools were used, as well as polishing. He also considered that several stone masons, perhaps even from various cultural groups, were involved in the manufacturing of the statue. Some scholars even considered the Hirschlanden warrior to be an imported semi-manufactured kouros from Greece, which was modified by a Celtic sculptor, or that the stone mason was a person who crossed the Alps to work on this sculpture [9, 63]. J. Röder also observed the use of multiple tools, such as a pick, pointed and flat tools and furthermore polishing, on the stelae from Stammheim, Holzgerlingen and especially from Steinenbronn [95].

As evident from the above discussion, important issues such as the existence of the earliest Central European monumental sculpture, lacks up to now a complex study focussed solely on technological aspects. So, the present article, with the results of traceological research on the Early La Tène statue, and fragments of at least three more statues, from the Glauberg, will open up this unsolved question.

**Fig 1:**
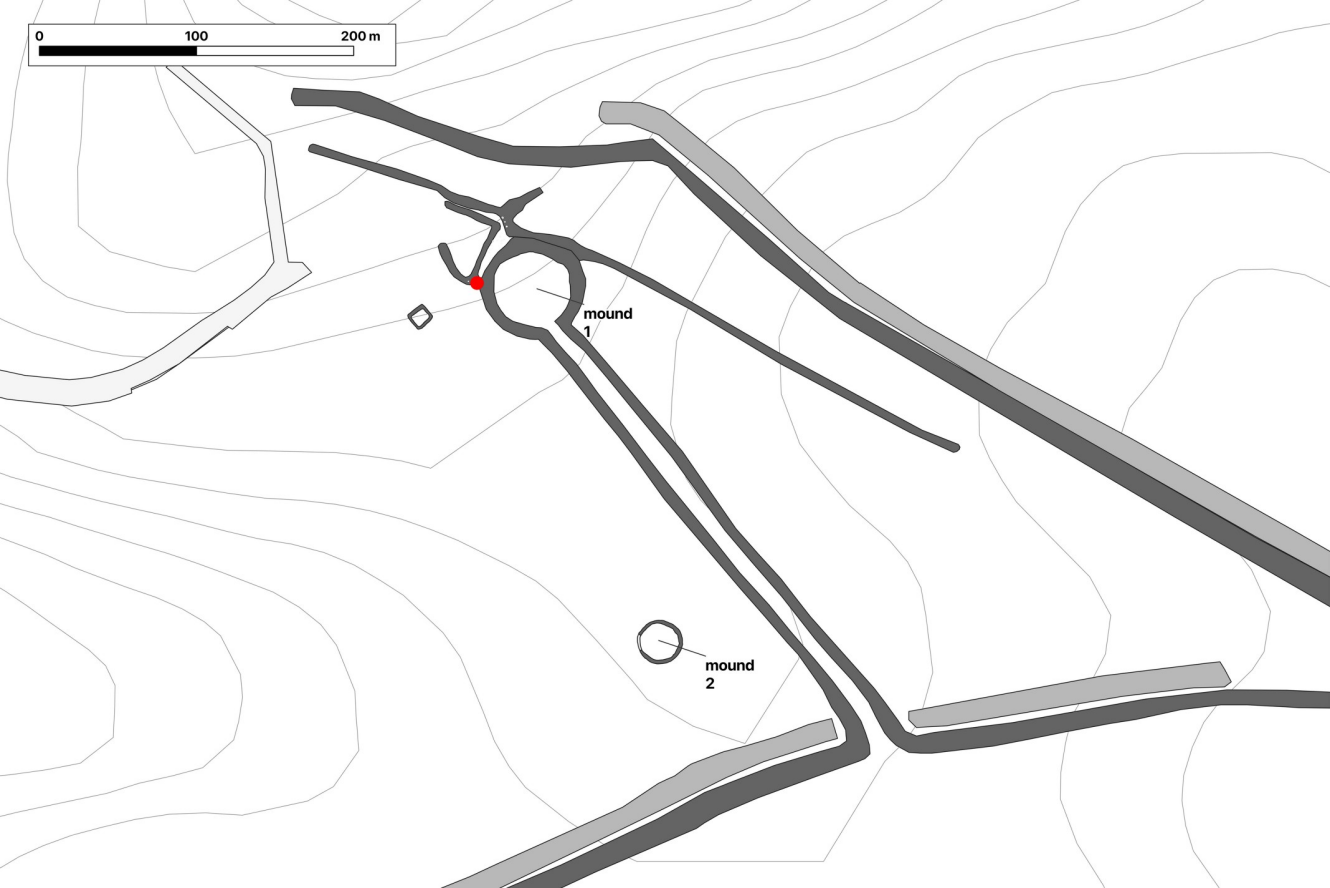
Map indication of the findspot of the complete statue and the fragments from at least 3 other sandstone statues in a ditch around burial mound 1 from the Glauberg (graphic: A. G. Posluschny, Keltenwelt am Glauberg).

**Fig 2:**
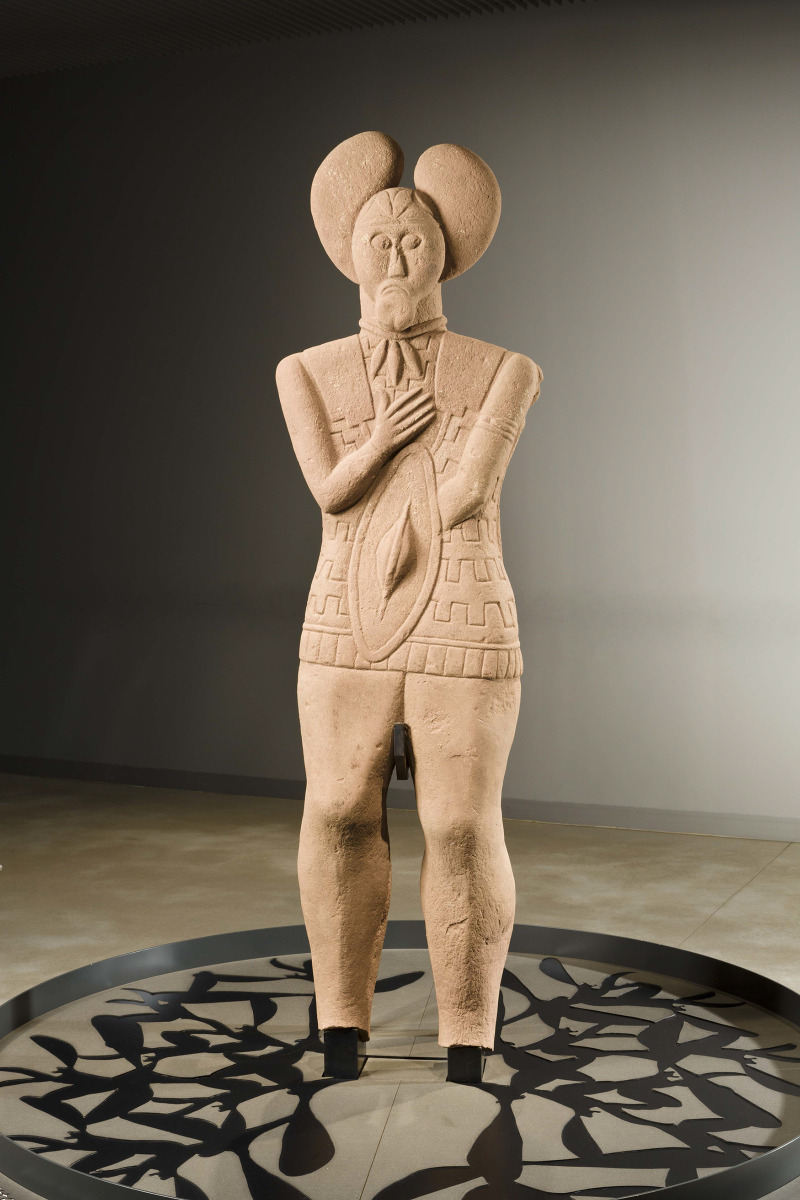
Sandstone statue (statue 1) from the Glauberg (photo: P. Odvody, hessenARCHÄOLOGIE).

A complete sandstone statue (statue 1) of an Early La Tène warrior, priest, druid or “prince” (or a combination of those statuses) was found in an annex to the ditch (Fig 1) surrounding the burial mound with the two main graves from the Glauberg [96]. The statue was complete preserved (Fig 2), missing only its feet and the base, and as such it is by far the best preserved and most elaborate life-size statue of the La Tène period north of the Alps. Some of its features, like the leaf cap (“mistletoe cap”), a necklace, a finger ring, a bracelet, a sword and a shield, have been found as grave goods of the ‘princely’ burial 1 in the nearby mound.

While the sword, the shield and the ornaments on the leaf cap clearly show a Celtic, Early La Tène origin, its cuirass is very obviously influenced by a Greek or Etruscan linothorax. A good comparison is the linothorax worn by the so-called Mars of Todi [97].

**Fig 3:**
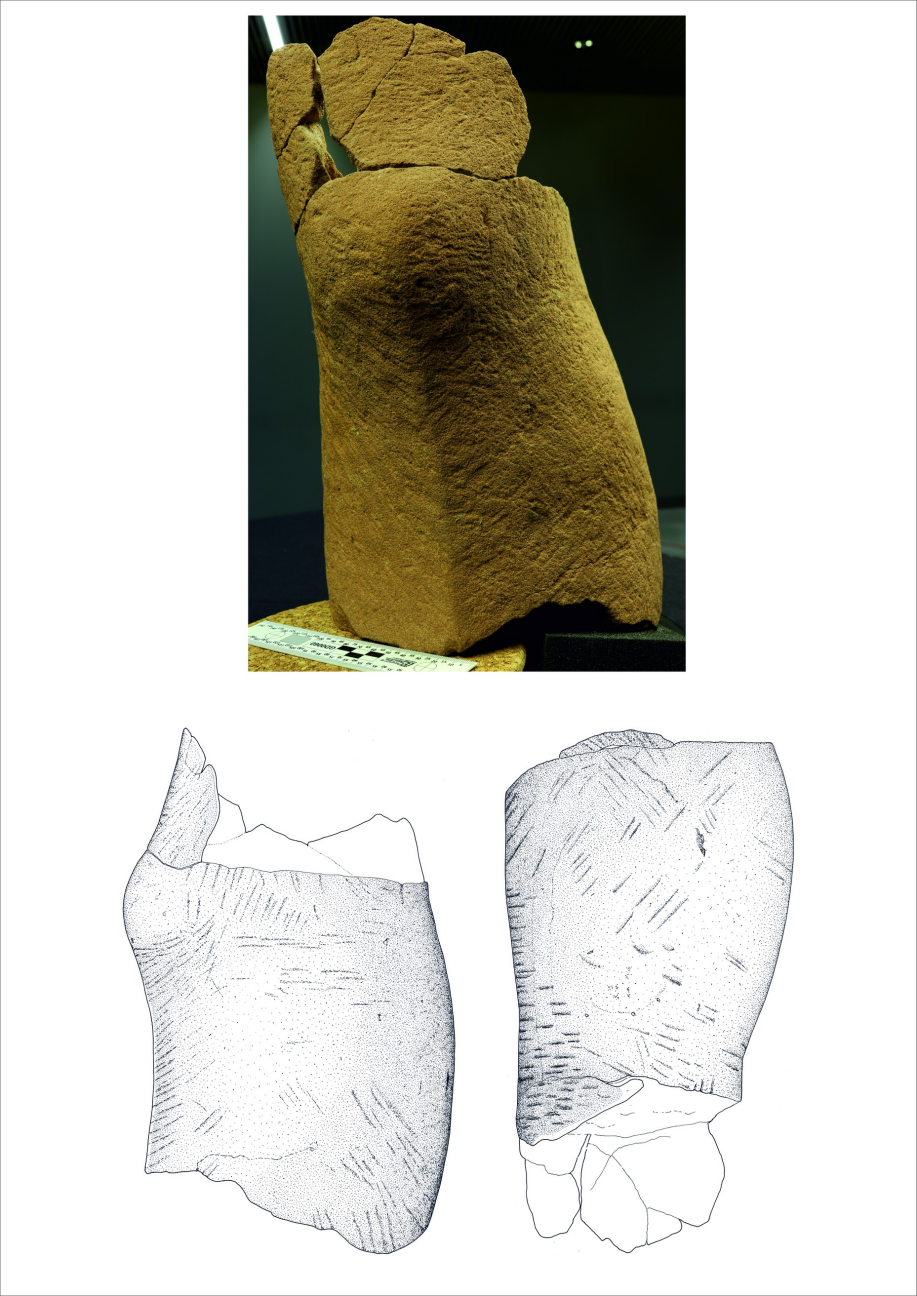
Fragment (leg) of a sandstone statue (statue 2) from the Glauberg (photo: F. R. Václavík; drawing: Pilar Rispa).

**Fig 4:**
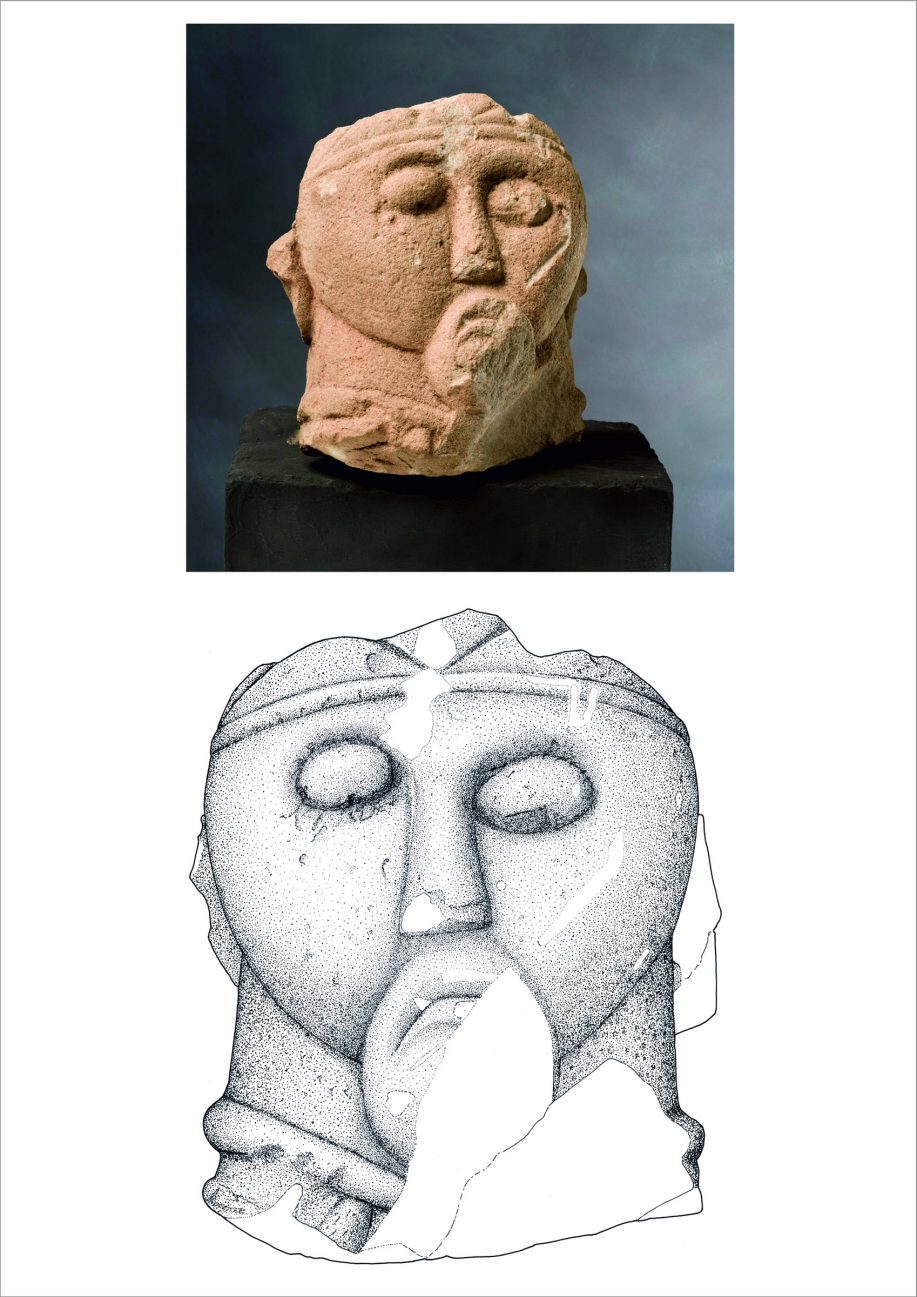
Fragment (head) of a sandstone statue (statue 3) from the Glauberg (photo: P. Odvody, hessenARCHÄOLOGIE; drawing: Pilar Rispa).

The discovery of the complete statue in 1996 was a sensation, but it was not the only find of that kind from the Glauberg. Scattered in the same ditch were the fragments of further statues that had been almost totally destroyed already in ancient times. Due to the fact that several fragments of a head have been found, and that sandstone of different colours was used, it is clear that the 130 remaining fragments represent at least three more statues; taken together, there are therefore remains of at least four similar, but not identical, statues from the Glauberg. For our analyses we have chosen three objects from the Glauberg: The complete statue 1 (Fig 2), the leg fragment of statue 2 (Fig 3) and a head fragment from statue 3 (Fig 4).

## 2. Method

Our investigaions used the methods of traceology, entailing the study of the working traces left on the object (mechanoscopy) and XRF measurement of the surface of the sculpture to detect the use of the iron or bronze tools (analytic traceology).

Mechanoscopy identifies the traces of the tools on the object. These traces or their dynamics may indicate the shape of the blade of the relevant tool, and may also contribute to the overall reconstruction of the working procedures of the stonemason or sculptor.

The initial phase consisted in creating digital models of the relevant stone pieces. We used a photogrammetric method for this purpose. Then, individual traces were localised on the model, depicted in orthogonal view and exported as.txt and.tif files. They were then transformed into a hypsometric model. A hypsometric model can generate both the transverse as well as the longitudinal traces of the tool. The shape of the blade is then given by the transverse section of the trace, whereas the longitudinal characteristics of the trace may define the trajectory of the passage of the tool or its dynamics.

The specified methods, which are exclusively non-destructive, have already been used with positive results during research on the Etruscan quarry of Cava Maggi, near the necropolis of Monterozzi in Tarquinia [95] as well as for the research of the stone head from the famous Iron Age hillfort Závist near Prague [32].

The analytic part of the method entails the identification of the residues of the abrasion or microscopic splinters from the tool used during work on the stone, and allows the identification of the metal used in the manufacture of the tool. Each tool becomes abraded in the course of its use. These splinters or abrasions remain in the light matrix of the stone. The metals in these splinters may be identified in the stone, according to the following processes. Sedimentary rocks include voids of varying size and shape, which are not filled with a solid phase. The porosity of the rock represents an important parameter influencing the transfer of the liquids in the rock [98]. The change in the concentration of ions and the humidity gradient relative to the surface causes a diffusion flow of the liquid into the interior of the stone structure. This physical phenomenon is accentuated by two other processes, namely by thermodiffusion, when the heat affecting the surface of the stone influences the penetration of the liquid to the interior, and also by proper liquid viscosity.

Atmospheric deposition causes the degradation of the metallic splinters left on the worked surface by the stonemason´s tool. These splinters may be dissolved and the metal they come from may be transferred as ions to aqueous solutions. These solutions may, as a result of the above described processes, migrate into the internal rock structure and cause the transfer of metallic ions under the stone material surface. The ions may, under the changing of the physical/chemical conditions, precipitate from the solutions again.

A second possibility is the migration of the microscopic splinters from the metallic tool, however, this variant is only possible in case of an adequate pore size, respectively of their diameter. In this case, the splinters move also to the interior of the stone.

**Fig 5:**
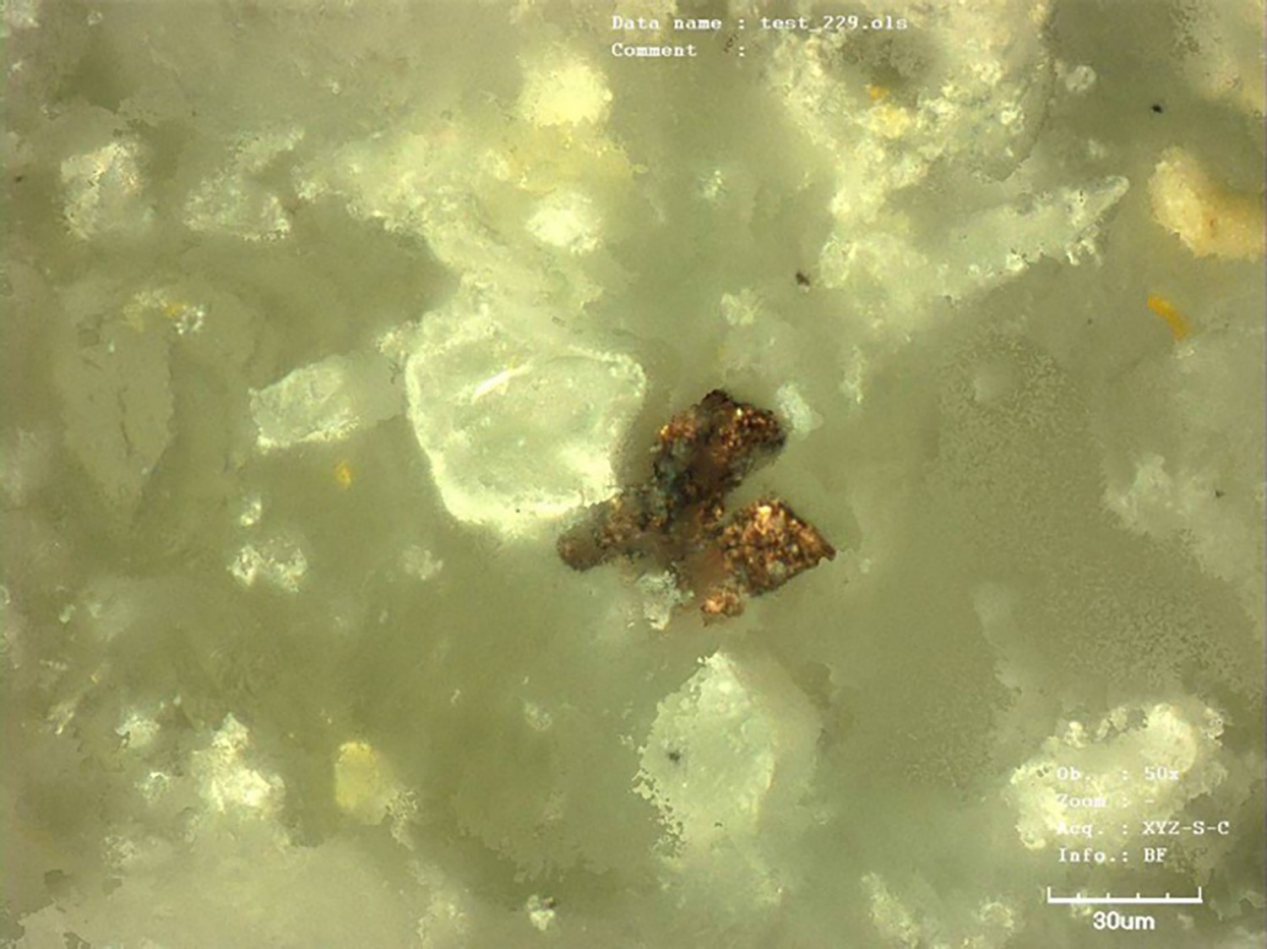
Experimental working up the surface of the sandstone block by the tool made of copper. Microsplinter of the copper from the tool in the structure of the worked surface (photo: M. Cihla).

**Fig 6:**
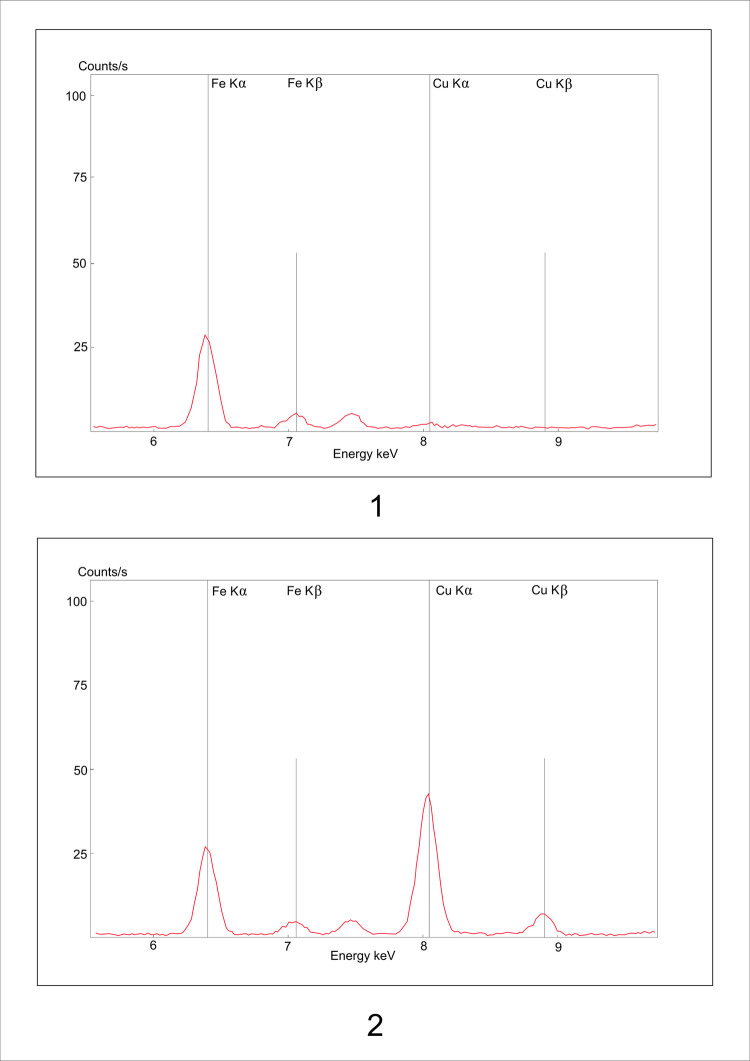
Experimental XRF measurement of the unworked (1) and worked (2) surface by the tool made of copper. The picture (2) shows a significant increase of the presence of the copper on the surface-red line (author: M. Cihla).

**Fig 7:**
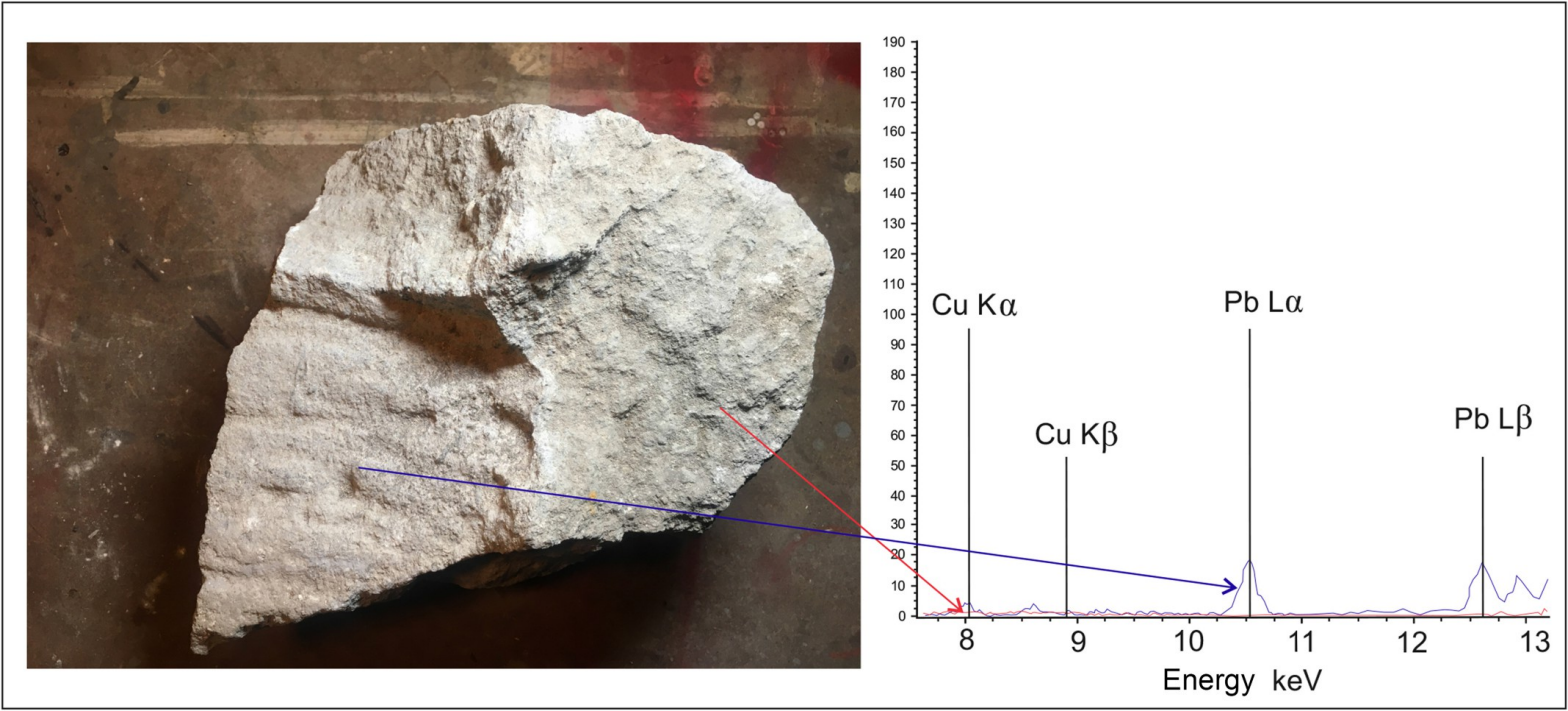
XRF measurement of the sample of the historic surface from the 4th century BC (interior of one of the tombs in the necropolis of Morre, Tarquinia). 1 non worked surface (red line), 2 worked up surface by the axe (grey line). The worked up surface shows a significant increase of the amounts of the copper and lead (photo and graph: M. Cihla).

The fact that the working of the stone surface leaves the metallic splinters (from the tool) in the light stone matrix was verified by an experiment. We have worked the surface of the sandstone block with a copper chisel. The copper splinters left in the stone were documented microscopically (Fig 5) as well as analytically. The surface of the block was XRF measured before and after working of its surface. The measurement after the surface working indicated a significant increase in copper presence (Fig 6:1–2). We have repeated the same analysis with more examples of historic worked surfaces, all with similar results (cf. Fig 7).

We call this procedure the “differentiation method”. It is based on the identification of the difference between the values of metals detected on the worked and unworked parts of the object. The basic criterion required for succesful results is the existence of worked, as well as unworked surfaces on the researched object. Unworked surfaces or parts may provide the information on the original composition of the object, whereas the worked areas are contamined by elements coming from the metal of the tool used by the stonemason.

When choosing the measuring properties of the device, we selected the mode identifying only the heavy metals and eliminating the light elements. Searching for the most suitable approach, we used experimentally the geological modes, interpreting all elements on the object´s surface. The mode “alloy”, specially designed for the detection of metals, was finally evaluated as the best option, since it is capable to reveal a real maximal difference between heavy elements on the worked, as well as unworked surfaces.

It is very important to take into account the possibility of contamination of the researched surface, for example by the surrounding soil etc. Thus, the most advantageous situation is when both the source material and the archaeological context are known. However, the contamination should cause the alteration of the values of relevant elements both on the worked as well as unworked surface. Thus, in cases when there are significant differences in the quantities of relevant elements on the worked compared to unworked surfaces, the possibility of contamination may be excluded.

The amount of particular element in the light matrix of the stone may also be affected by the location of the researched object in the exterior or interior. This was clearly prooved during the abovementioned research of the chamber quarry Cava Magi in Tarquinia, where the amounts in the working traces of individual tools were in many cases really high [95]. Conversely, with the exposure of the studied object to the external influences, such as the weather, the possibility of the identification of the relevant elements decreases.

For the documentation of the stone fragments as well as the complete statue, a Canon 6D Mark II camera with a Canon 35 mm lens was used. The photographic documentation was carried out using a tripod and with the following settings: ISO 160, shutter f/11, focal length 35 mm; and without a tripod: ISO 320, shutter f/5, focal length 35 mm. The 3D model was generated by overlaping the individual pictures by more than 50%. Table 1 shows the number of photographs used for each 3D model.

**Table 1:** Images used for the photogrammetric modelling of statue 1 from the Glauberg and some of its fragment.

Object	Images taken
Complete statue (statue 1)	2300
Leg fragment (statue 2)	320
Head fragment (statue 3)	180

The 3D models were generated using the software AgisoftPhotoscan Version 1.4.5 build 7354. For generating the topographic model, the software Global Mapper Version 18 and RhinoCad 5.0 were used.

XRF measurement was carried out using the device Vanta Olympus, set on the mode “alloy plus” intended exclusively for the detection of heavy elements. The detection of the light elements was intentionally eliminated, providing for the possibility of the presence of corrosive processes. The mode “geo” is also available on the used device, which is intended for measurements of the element compositions of rocks, including light elements.

## 3. Results of traceology and XRF analyses

### Mechanoscopy and XRF measuring of the fragment of the head–Statue 3 (Figs 8 and 9 and Fig 10:1–8; Table 2:ID1–8)

**Fig 8:**
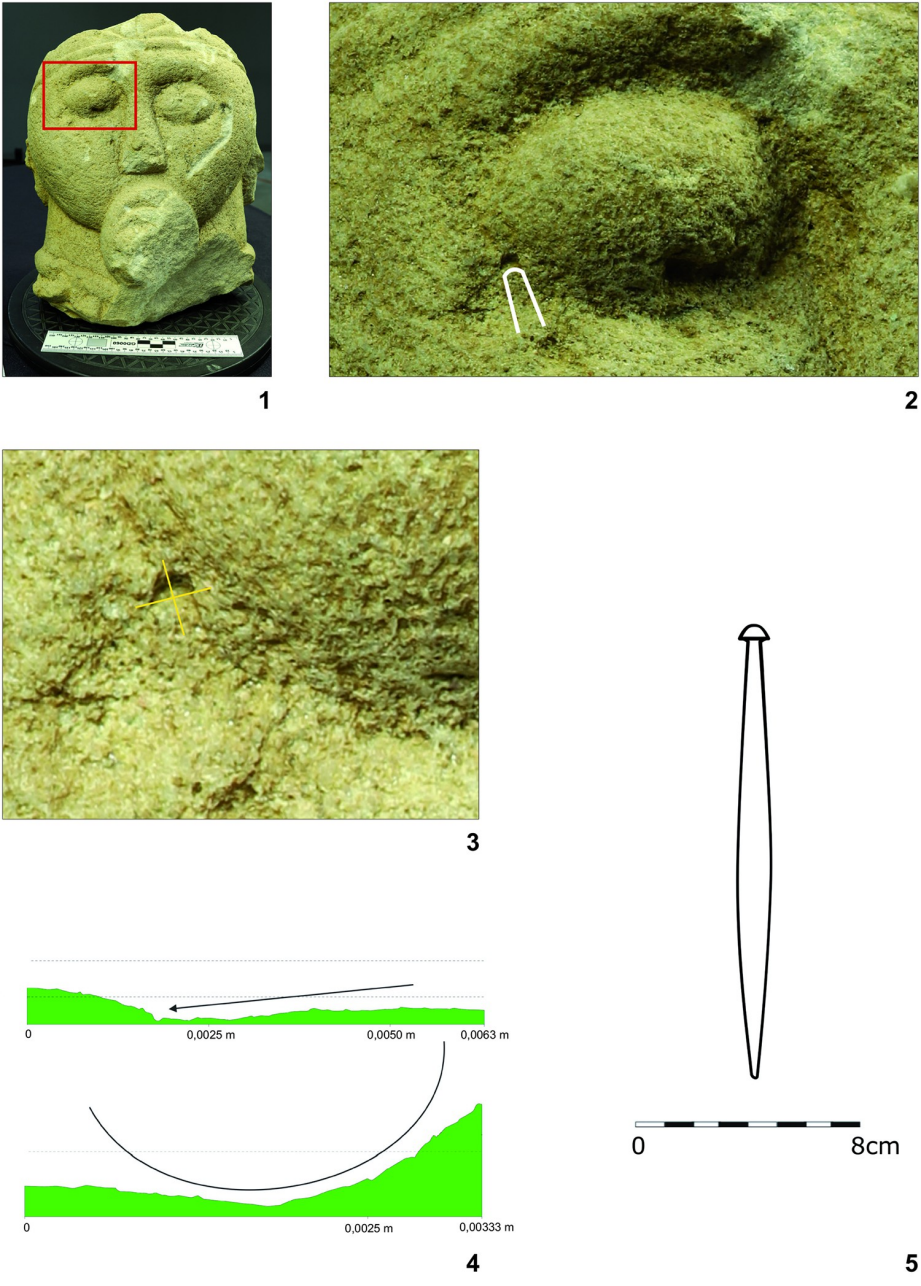
Fragment of the head from (statue 3) from Glauberg. 1–2 the area of the eye with clearly visible trace (in red square, other visible scratches are modern); 3–4 the longitudinal and transverse sections of the trace and derivation of the shape of the blade of the tool (black line); 5 the hypothetic shape of the tool as reconstructed from the longitudinal and transverse sections (author: M. Cihla and F. R. Václavík).

**Fig 9:**
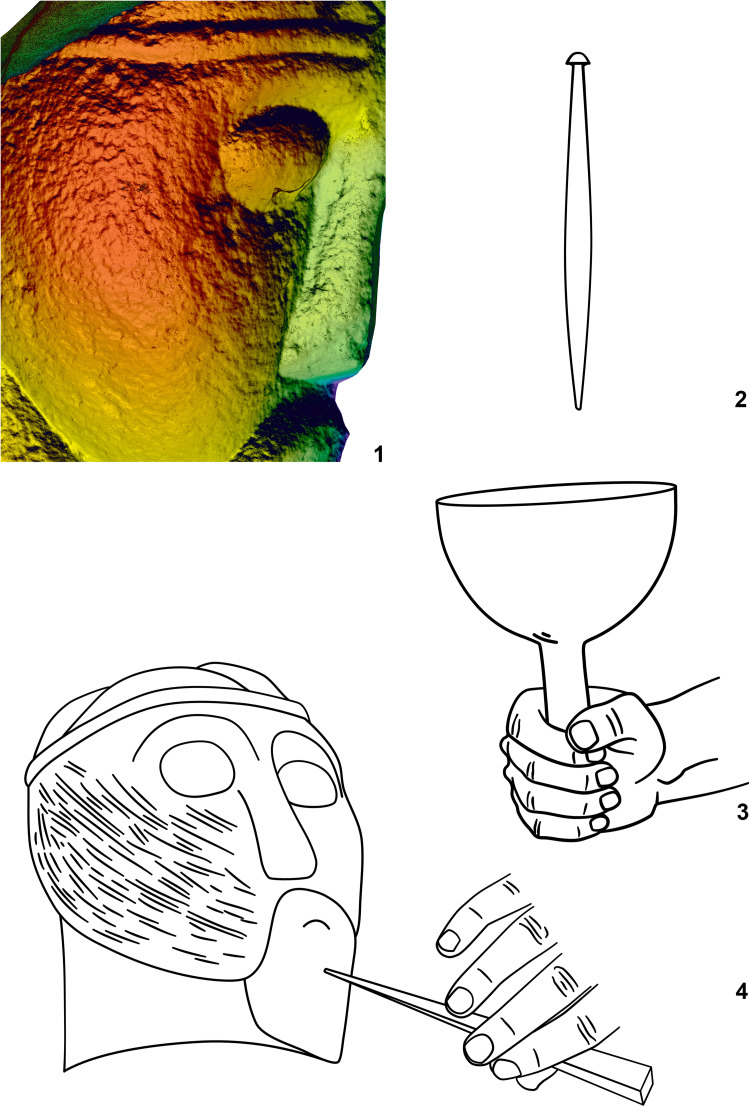
Fragment of the head (statue 3) from Glauberg. 1 the digital model shows individual traces; 2–3 the reconstructed tool and the mallet; 4 reconstruction of the finishing of the surface in parallel rows (author: M. Cihla and F. R. Václavík).

**Fig 10:**
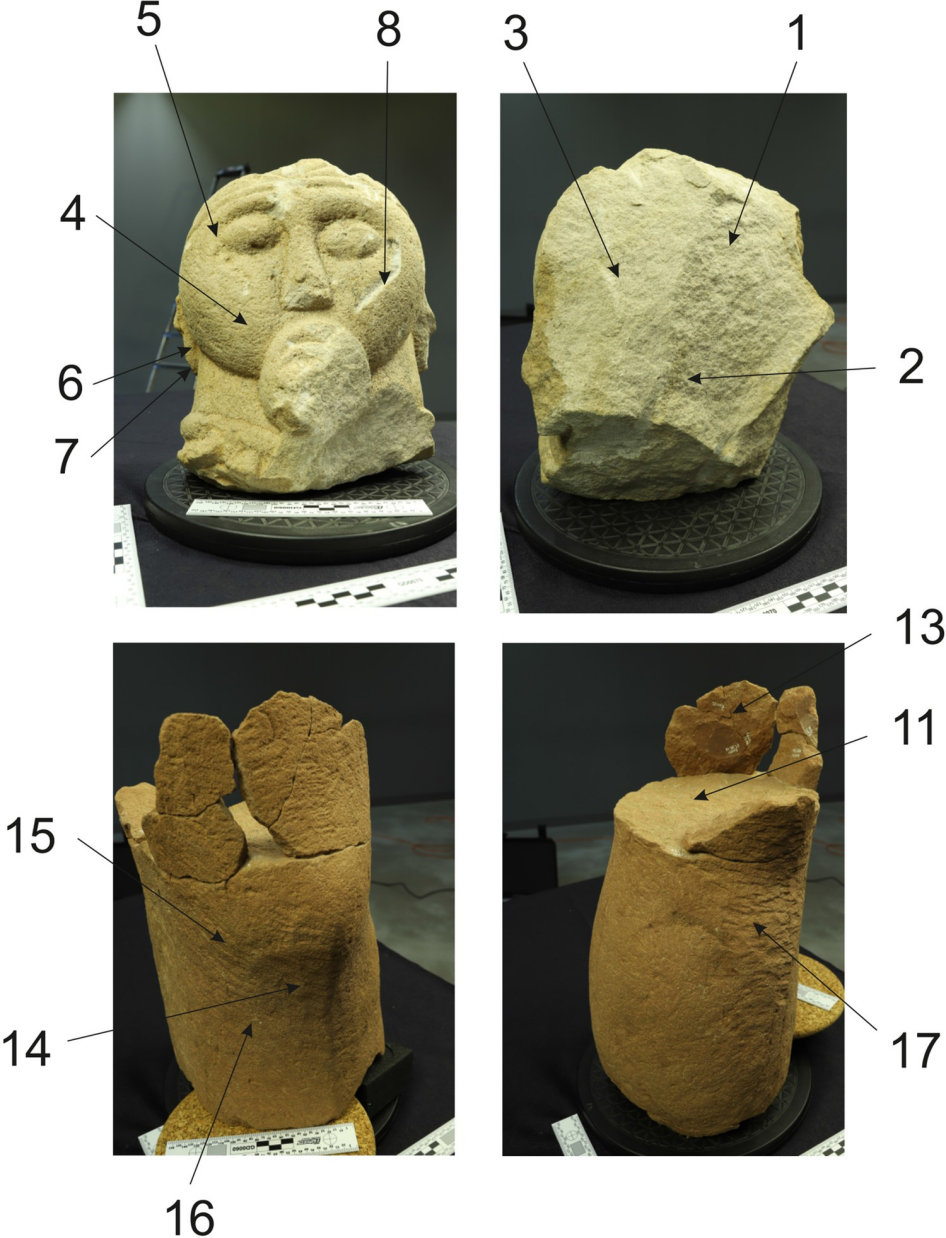
Points of the X-ray fluorescence measurements (author: M. Cihla and F. R. Václavík).

**Table 2:** Results of the X-ray fluorescence measurements in individual points.

ID	Ti	V	Cr	Mn	Fe	Co	Cu	Zn	Zr	Sn	Sb	Pb	Bi
1	16.35372	4.976451	0	0.854994	51.55732	0	0	1.197379	4.677974	0	13.27718	7.104983	0
2	14.86509	0	0.443218	1.083048	69.76807	0	0	1.849167	6.089571	0	0	5.901832	0
3	15.27824	2.859788	0	0.819844	66.23821	0	0.674319	1.739997	5.285533	0	0	6.746139	0.357937
4	8.842971	0.691638	0.279246	1.112898	76.53636	0	0	1.331147	3.897214	4.453607	0	2.651188	0.203731
5	6.256348	0.929693	0.247752	6.426178	73.11222	0.37359	0	0.873208	1.642952	2.107899	5.807017	2.223143	0
6	6.093679	0	0	1.929755	81.94778	0	0.707415	1.718253	3.501754	0	0	3.774016	0.327352
7	9.081718	0	0.358462	1.022128	74.20279	0	0.194403	0.710671	2.689896	3.916088	5.419612	2.404231	0
8	5.390184	0.613242	0	1.384021	81.62707	0	0	3.425355	2.452843	0	0	5.107288	0
11	4.662187	0.389712	0	1.064054	81.28159	0	0.186248	2.15661	1.872891	0	5.958956	2.427749	0
13	4.586903	0.647993	0	0.613817	83.00669	0.620332	0.157271	1.289986	1.85922	1.941103	3.241075	1.898219	0.137394
14	4.732289	0.344872	0	2.512104	82.03642	0	0	2.282357	4.865582	0	0	3.226375	0
15	5.354	0.414566	0.190312	2.337554	76.74327	0	0.203938	4.840983	3.03772	0	4.193605	2.684052	0
16	5.667235	0	0	0.86369	83.83937	0.412442	0.27771	1.802592	2.973649	0	0	4.163311	0
17	6.845317	0	0	1.070245	74.10439	0	0.64324	2.021316	4.176597	0	7.037431	4.10146	0
20	6.523943	0.656251	0.252753	2.266226	81.99346	0	0	0.766556	2.120584	0	3.282462	2.137764	0
21	6.501013	0.317066	0.214392	2.731366	80.2737	0.326425	0.158235	0.589198	1.953722	1.74645	3.701833	1.486595	0
22	4.834113	0.463702	0.130838	0.984495	85.52218	0	0	0.657591	1.858292	1.448234	2.636161	1.318489	0.145901
23	5.493049	0.389267	0.203072	2.305829	82.80162	0.708959	0	2.86114	2.737851	0	0	2.269589	0.229627
24	6.2774	0	0	3.412161	83.99052	0.346727	0.270009	0.918875	2.65712	0	0	2.023796	0.103398
25	6.786639	0.537924	0.166847	3.760789	80.33519	0.619562	0.196572	0.468583	2.591686	0	2.875359	1.488074	0.172772
26	5.251173	0.376495	0.259849	0.67902	84.17448	0	0	1.027717	5.087084	0	0	2.94641	0.19777
27	4.72333	0.906753	0.16735	4.28767	76.28404	0.555344	0	2.849244	2.954179	0	3.905807	2.943568	0.42271
28	5.809768	0.408364	0.141275	1.453458	86.26076	0	0	1.1903	2.628636	0	0	2.107445	0
29	8.136765	0.798879	0	1.004179	81.78372	0	0	1.412767	4.328111	0	0	1.843579	0.691997
30	6.954234	0.669336	0	1.742477	81.2692	0.493935	0	0.756048	2.604312	0	3.328286	1.961547	0.220621
31	6.841107	0.336508	0.086834	1.608652	83.41927	0	0	1.423477	2.666868	2.031986	0	1.585295	0

The facial part of the head shows clearly visible secondary impacts (Fig 8:1), not connected with the manufacturing activities, but also traces of the sculptor´s tools used in the final treatment of the surface (Figs 8:2–5 and 9:1). Unfortunately, no traces connected with the previous or primary sculpting phases were found.

The sculpting traces found in the facial part and in the area of the eyes are similar to each other. They are rather dynamic, showing certain power of the strikes, as individual traces are aligned behind each other (Fig 9:4). Their shape in the longitudinal direction is straight (Fig 8:4). All these traces provide evidence about the handleless tool (a tool without a wooden handle), very probably a kind of chisel. The transverse section of the blade of the tool is of a rounded form and the width is up to 3 mm (Fig 8:4).

The described tool was used for making the “almond-shaped” eyes, as well as for “finishing” the surface of the face (Fig 8:1). This finishing process was carried out by making parallel rows from the beard up to the diadem. Each row is produced by a series of short strikes of the chisel. The overall length of the tool can be only estimated; nevertheless, based on the relative dynamics of the strikes, we may consider that it was most likely around 15–20 cm.

As for the XRF analysis of the surface of the head (Fig 10: 1–8; Table 2:ID1–8), as well as of other fragments including statue 1, unfortunately we do not know the values of the individual elements in the original stone, since we do not know where exactly the stone for the sculpture was quarried. Nevertheless, we have samples of local sandstone, coming from the site of Bleichenbach, some 6 km northeast of the Glauberg. Sand stone has been quarried here up to the 20^th^ century AD and the stones used for the Glauberg statues could have been sourced here or at least from the same bank of sand stone, stretching from here further northwest and southeast. The values of individual elements of the composition of these two samples (Table 3) may significantly contribute to the correct evaluation of the measured results. They generally correspond (with little variations, for example lower amount of the iron in the material of the head of statue 3) with the measurements from the stone of all statues. However, because our assumption about the possible origin of the source stone for all researched sculptures and fragments is not completely sure, we must focus our attention also on the places on the fragments which were not worked up, most obviously the fractures revealing the interior parts of the stone (Fig 10: 1–3). The measured values in these parts may provide an estimate of the values of the original source stone. However, also in this situation the hypothetical contamination of the broken away places (cf. supra) cannot be fully ruled out. For this purpose we have measured also the element composition of three samples of the soil, taken immediately from the surface of the statue 1 during the excavation (Table 3).

**Table 3:** Results of the X-ray fluorescence measurements of two samples of sandstone from Bleichenbach and samples of the soil immediately surroundning the statue 1.

Sample	Ti	V	Cr	Mn	Fe	Co	Ni	Cu	Zn	As	Zr	Sn	Sb	Pb	Bi
stone A ID1	4.5	0.1	0	0.15	82	0	0.55	0.71	0.52	0.04	5.72	0	3	1.03	0
stone A ID2	4.3	0.2	0.1	0.26	83.66	0.73	0.5	0	0.64	0.05	4.36	4.4	0	0.86	0
stone B ID 1	4.6	0.13	0.07	0.48	82.12	0	0.47	0	0.62	0.06	4.93	3.4	1.8	0.63	0
stone B ID 2	5.2	0.1	0.14	0.16	84.9	0	0.68	0.75	0.7	0.08	2.94	0	2.2	0.9	0
stone B ID 3	5.8	0.1	0	0.13	75.2	0	0.51	0.91	0.55	0	10.43	4.5	0	0.95	0
stone B ID 4	6	0.1	0.06	0.16	80.3	0	0.55	0.76	0.54	0.06	9.44	0	0	0.99	0
soil sample 15 ID 1	4.54	0.12	0.15	1.48	83.48	1.06	0.66	0.32	0.59	0	4.91	0	1.4	0.6	0.23
soil sample 15 ID 2	5.51	0.14	0.18	1.56	80.04	1.08	0.5	0.29	0.52	0.005	7.38	0	0.9	0.6	0.18
soil sample 15 ID 3	4.84	0.14	0.14	1.82	82.5	1.01	0.54	0.28	0.55	0.014	4.79	1.7	0.9	0.53	0
soil sample 15 ID 4	5.17	0.13	0.17	0.92	84.94	0.56	0.55	0.37	0.61	0	5.03	0	0.5	0.67	0.12
soil sample 2 ID 1	5.09	0.12	0.14	1.46	81.82	1.05	0.56	0.31	0.56	0	6.34	0	1.4	0.62	0
soil sample 2 ID 2	5.12	0.12	0.15	1.15	81.94	1.09	0.59	0.33	0.58	0	6.14	0	1.4	0.55	0.2
soil sample 2 ID 3	5.26	0.11	0.15	1.38	81.36	1.13	0.58	0.36	0.63	0.011	6.49	0	1.1	0.62	0.23
soil sample 6.1 ID 1	5.59	0.12	0.18	1.32	79.61	1.07	0.5	0.3	0.53	0	6.42	2.1	1.3	0.55	0
soil sample 6.1 ID 2	4.66	0.09	0.16	1.39	83	1.15	0.58	0.3	0.58	0	5.71	0	1	0.58	0.22
soil sample 6.1 ID 3	5.05	0.17	0.14	1.47	81.02	1.03	0.54	0.32	0.55	0	5.78	2.3	0.7	0.62	0
soil sample 6.1 ID 4	5.5	0.16	0.17	1.39	79.66	1.14	0.53	0.28	0.53	0	6.13	2.1	1.3	0.58	0.19

The measuring of the broken part revealing the interior of the head (Table 2:ID 1–3) and the measurement of the surface worked up by the sculptor (Fig 10:4–7; Table 2:ID 4–7) showed certain differences, especially in concentrations of iron, manganese and tin. The measurement in the secondarily damaged (scratched) places (Fig 10:8; Table 2:ID 8) even revealed three times higher concentrations of zinc, than the measurements from the interior of the stone.

Thus, the results of measurements 1–8 (Table 2:ID1–8) indicate that the elements left on the surface probably come from the chisel used for working up the surface of the head fragment, which was most likely made of iron. The presence of tin in the surface measurements could indicate a particular contamination during the manufacturing process of the chisel. One may argue, that also the composition of the soil from statue 1 includes a not negligible amount of iron. However, the contamination of statue 3 by elements of the surrounding soil seems improbable, since the increase of iron in the working traces left by the tool is characteristic also by the increase of manganese and zinc or by differences in the amount of lead (Table 2).

On the other hand, the almost complete absence of copper, both in the interior of the stone and on the worked up surface, indicates that most likely no tools made of copper or bronze were used during the manufacturing process of the head.

### Mechanoscopy and XRF measurements on the leg fragment–Statue 2 (Figs 11–14 and Fig 10:11–17; Tables 2:ID11–17 and 3)

**Fig 11:**
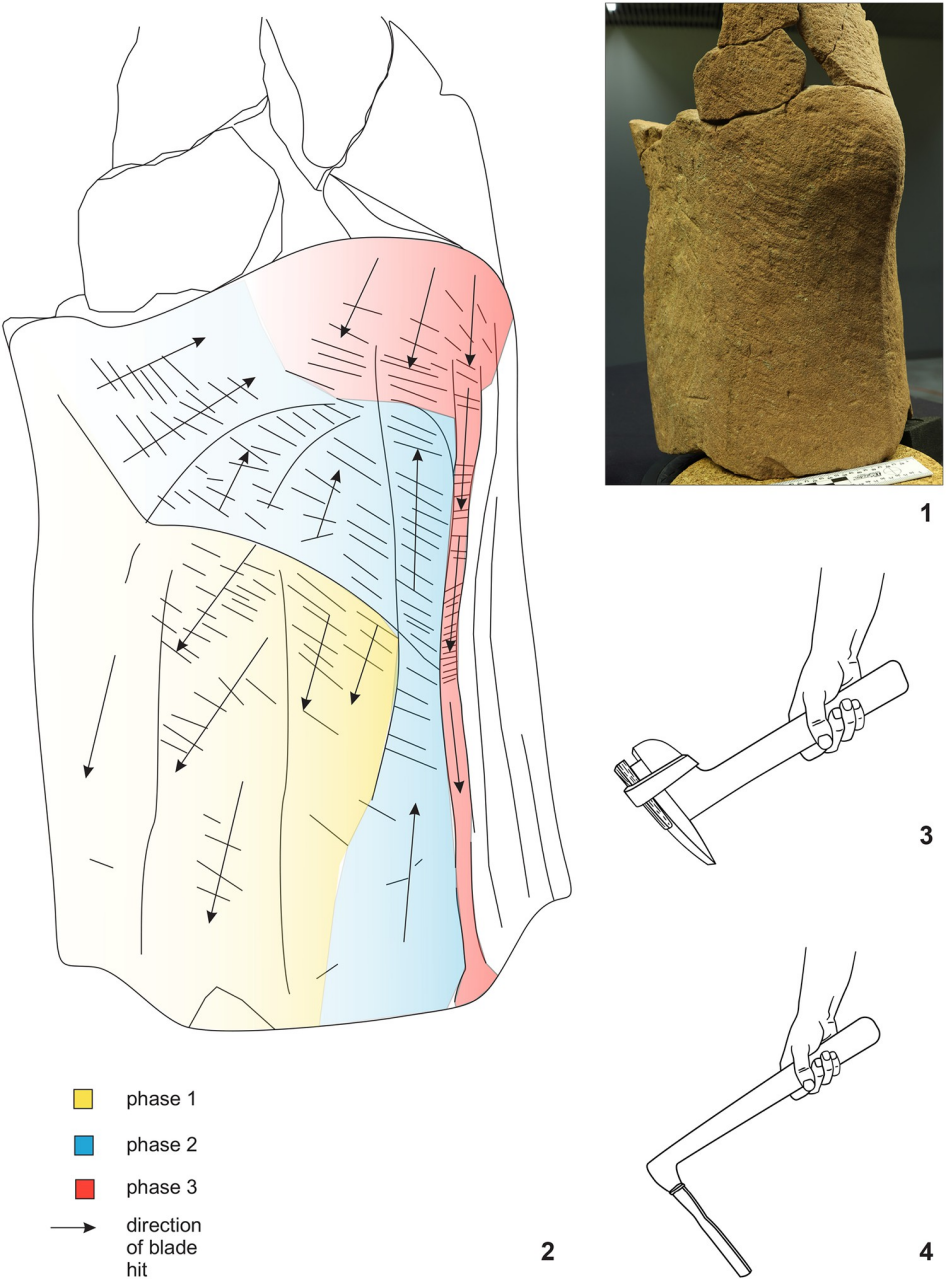
Fragment of a leg of the Statue 2 from Glauberg. 1 the fragment; 2 individual planes with the same direction of the hits of the tool; 3–4 hypotetical ways of the use of the relevant tool (adze) (author: M. Cihla and F. R. Václavík).

**Fig 12:**
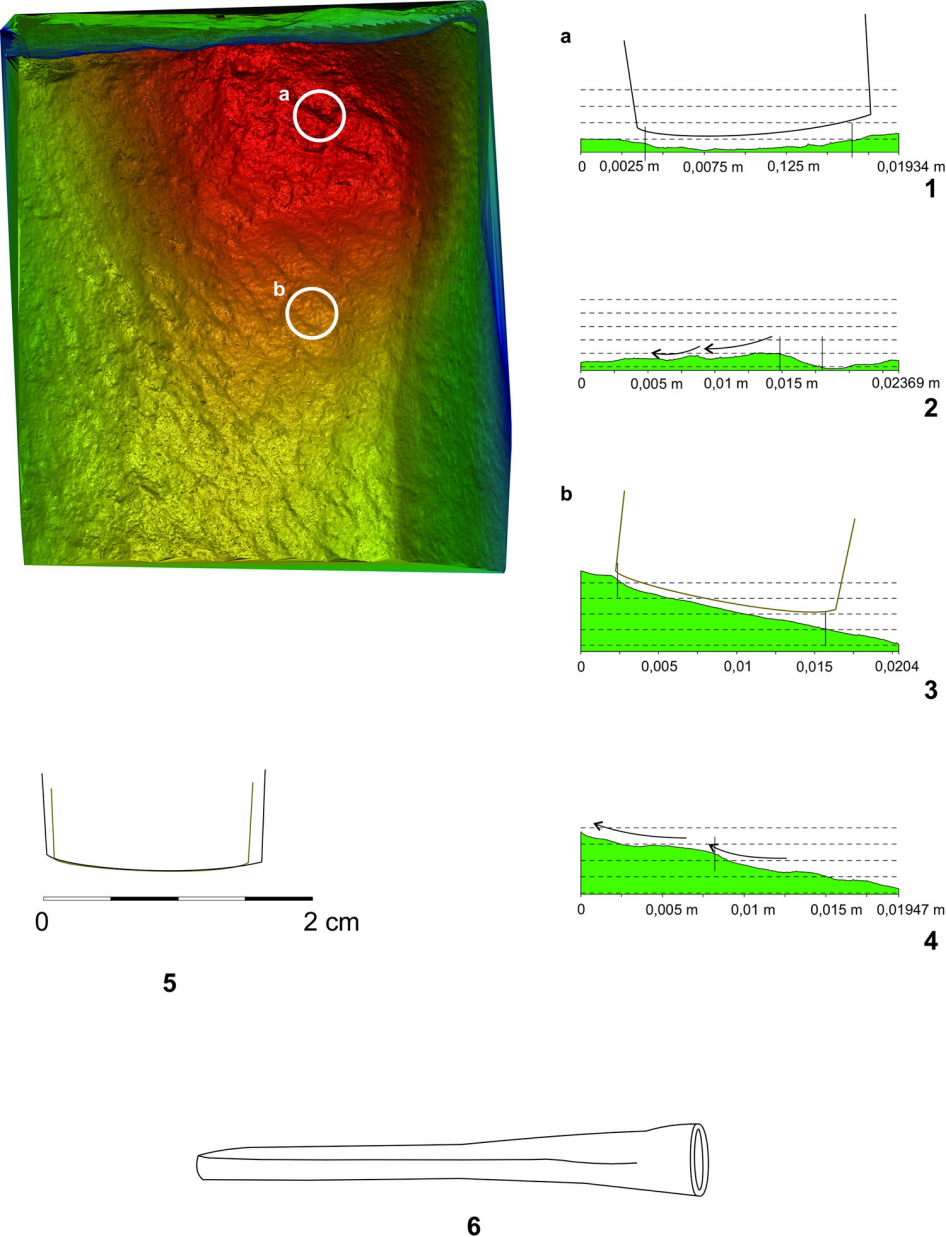
Fragment of a leg of the Statue 2 from Glauberg. 1–2 transverse and longitudinal sections of the identified trace (a); 3–4 transverse and longitudinal sections of the identified trace (b); 5 reconstruction of the identified blade of the tool as derived from individual traces; 6 hypothetical reconstruction of relavant tool (author: M. Cihla and F. R. Václavík).

**Fig 13:**
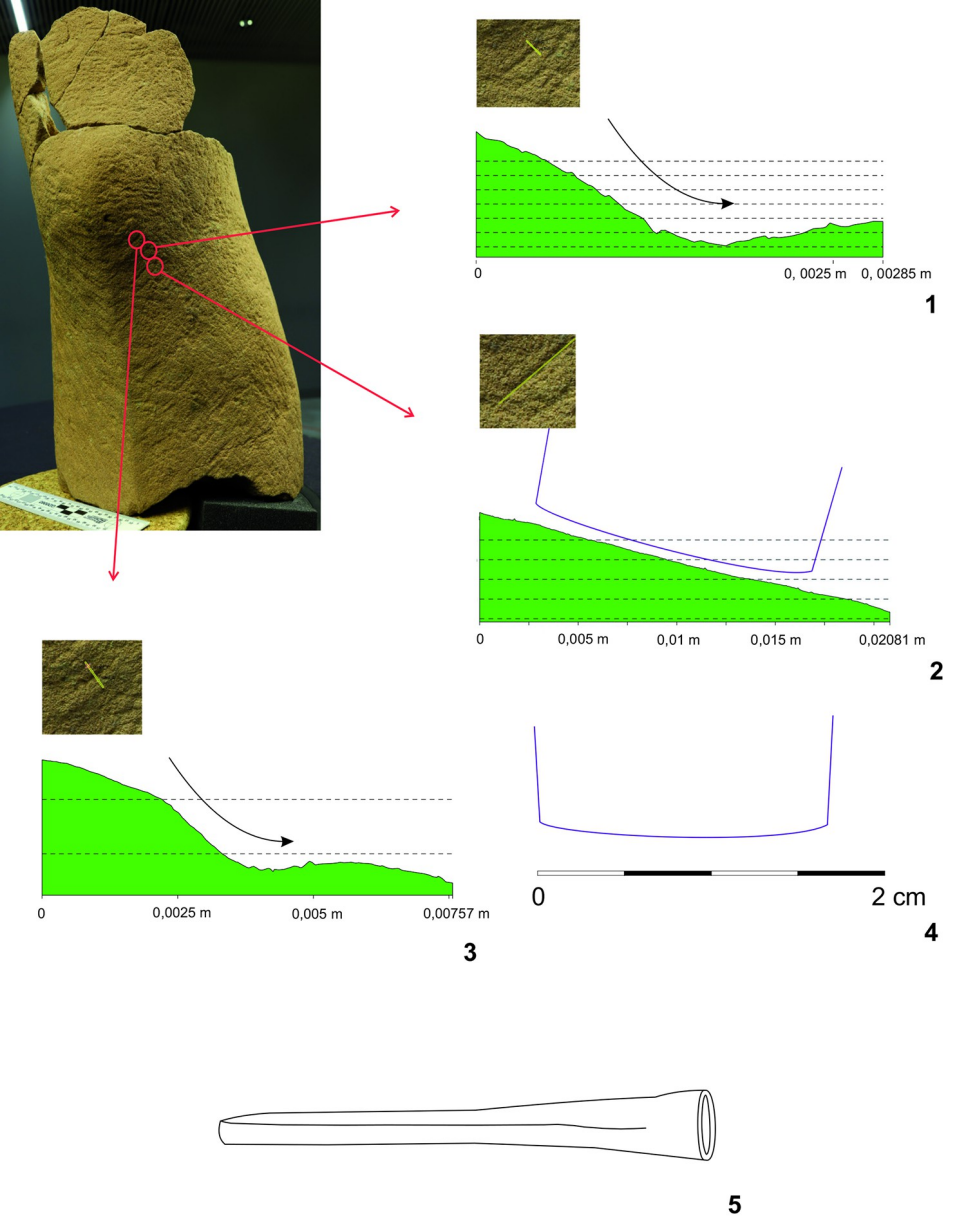
Fragment of a leg of the Statue 2 from Glauberg. 1 longitudinal section of the identified trace; 2 transverse section of the identified trace; 3 longitudinal section of the identified trace; 4 reconstruction of the identified blade of the tool as derived from individual traces; 5 hypothetical reconstruction of relevant tool (author: M. Cihla and F. R. Václavík).

**Fig 14:**
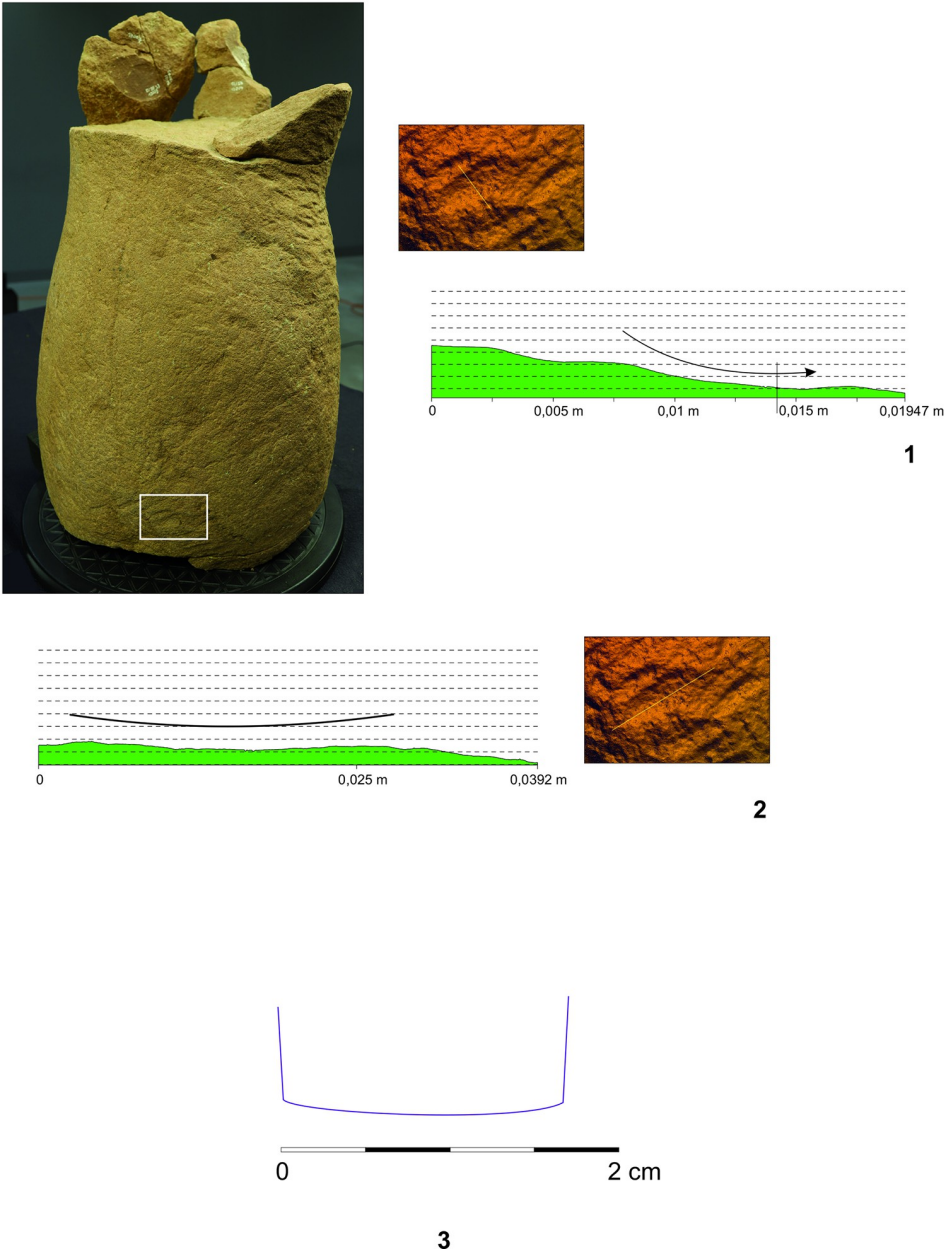
Fragment of a leg of the Statue 2 from Glauberg. 1 longitudinal section of the identified trace on the calf; 2 transverse section of the same trace; 3 reconstruction of the identified blade of the tool as derived from the trace (author: M. Cihla and F. R. Václavík).

The working traces are visible especially on the shin (Figs 11–14). The area of the calf does not show so many traces. All the traces identified here belong to one tool. Lengthwise, the cuts have a rounded shape (Fig 12:2, 4), what confirms the supposition of the tool with handle. The cut is also dynamic and proportional. It means that the trace of the blow may be identified in its overall length. We suppose that the traces were made by the transverse blade of an adze (Fig 12:5), with the sculptor facing the worked object and making circular movement by the tool, creating individual traces aligned in rows (Fig 11).

The blade of the adze was slightly rounded and its width reached ca. 1,7 cm (Fig 12:5, 13:4, 12:3). The body of the adze was perhaps slightly extended. Of course, it is not possible to reconstruct the whole shape of the tool, and in our reconstruction its attachment to the handle is hypothetical (Fig 11:3–4).

The basic form of the leg was shaped in planes, so the transverse section of the leg was of a polygonal form (Fig 11:2). Our reconstruction shows individual phases of working up the leg in zones with the same direction of strikes.

The XRF measurement of the fragment of the leg (Fig 10:11–17; Table 2:ID 11–17) showed a distinct difference from the results of the head fragment, especially in the increase of the values of titanium, zinc, and copper on the worked surface (Fig 10:14–17; Table 2:ID 14–17). It is possible that such increase could indicate the abrasions of a tool made of a copper alloy. Thus, it is very tempting to suppose that the identified adze could have been produced from **bronze**. Nevertheless, this assumption cannot be fully confirmed, since the amounts of copper in the working traces on this fragment and in the natural soil from the site are comparable (Tables 2 and 3). Therefore, the contamination of the surface of the fragment by the copper from the surounding soil cannot be fully excluded.

As for the basic components of bronze, copper usually represents the principal metallic element (in tens of percents). However, the measurements on the worked surface (Fig 10:14–17; Table 2:ID14–17) showed that copper, abraded from the hypothetical bronze tool, was present only in substantially lower amounts (tenths of percent). Although we are still unable to explain this phenomennon, we identified it already several times in our research, for example in the Etrusco-Roman subterranean quarry Cava Magi (Tarquinia, Italy), where the abrasions of the bronze quarrymen´s tools were also sometimes characteristic by such low concentrations of copper [97].

### Mechanoscopy and XRF measuring of the complete Statue 1 (Figs 15–21 and, Fig 22: 20–31, Tables 2:ID 20–31 and 3)

**Fig 15:**
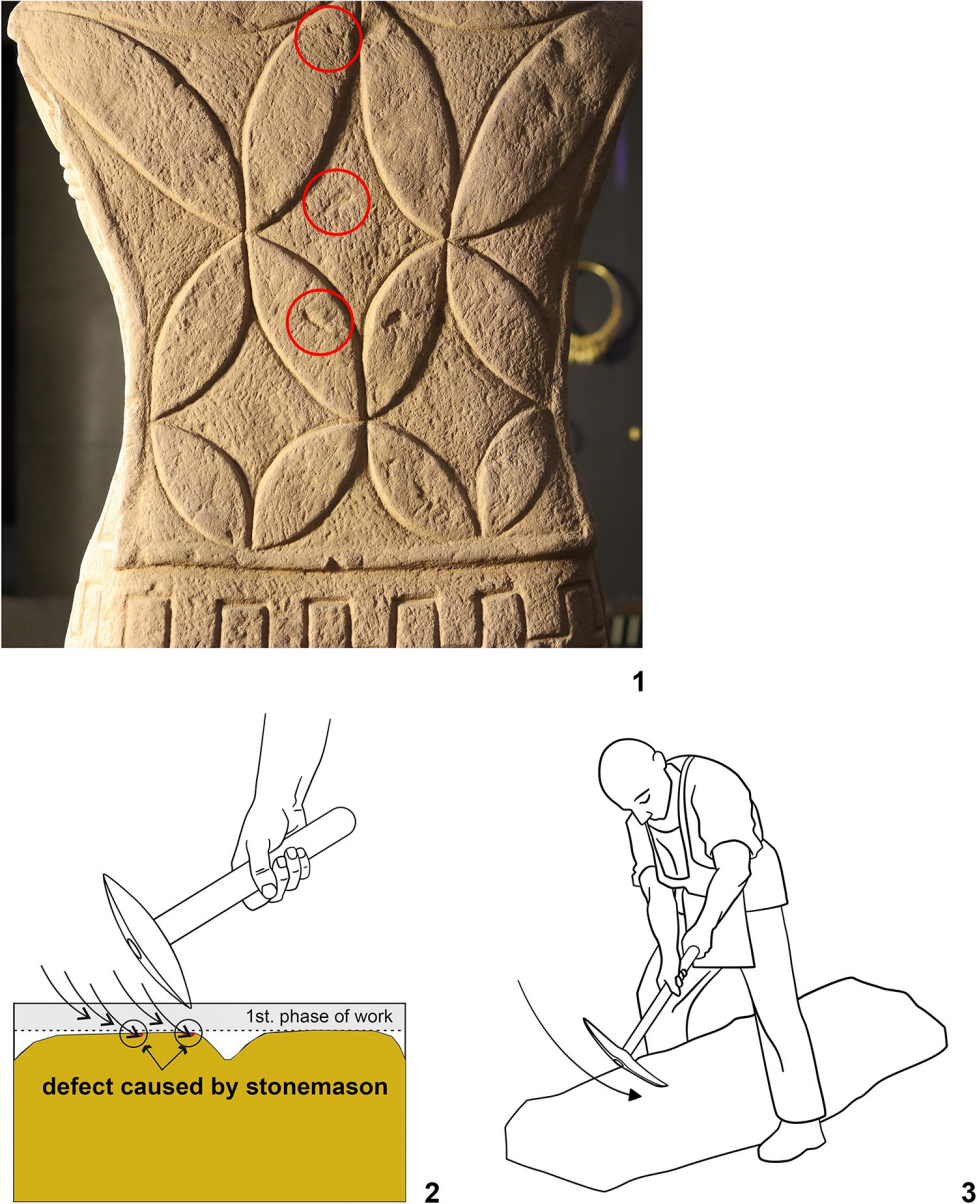
Statue 1 from Glauberg. 1 the traces of the pick (in red circles) on the sculpture´s back; 2 the scheme of the intitial phase of the working up the surface with a pick; 3 the position of the sculptor and the stele during the working with a pick (author: M. Cihla and F. R. Václavík).

**Fig 16:**
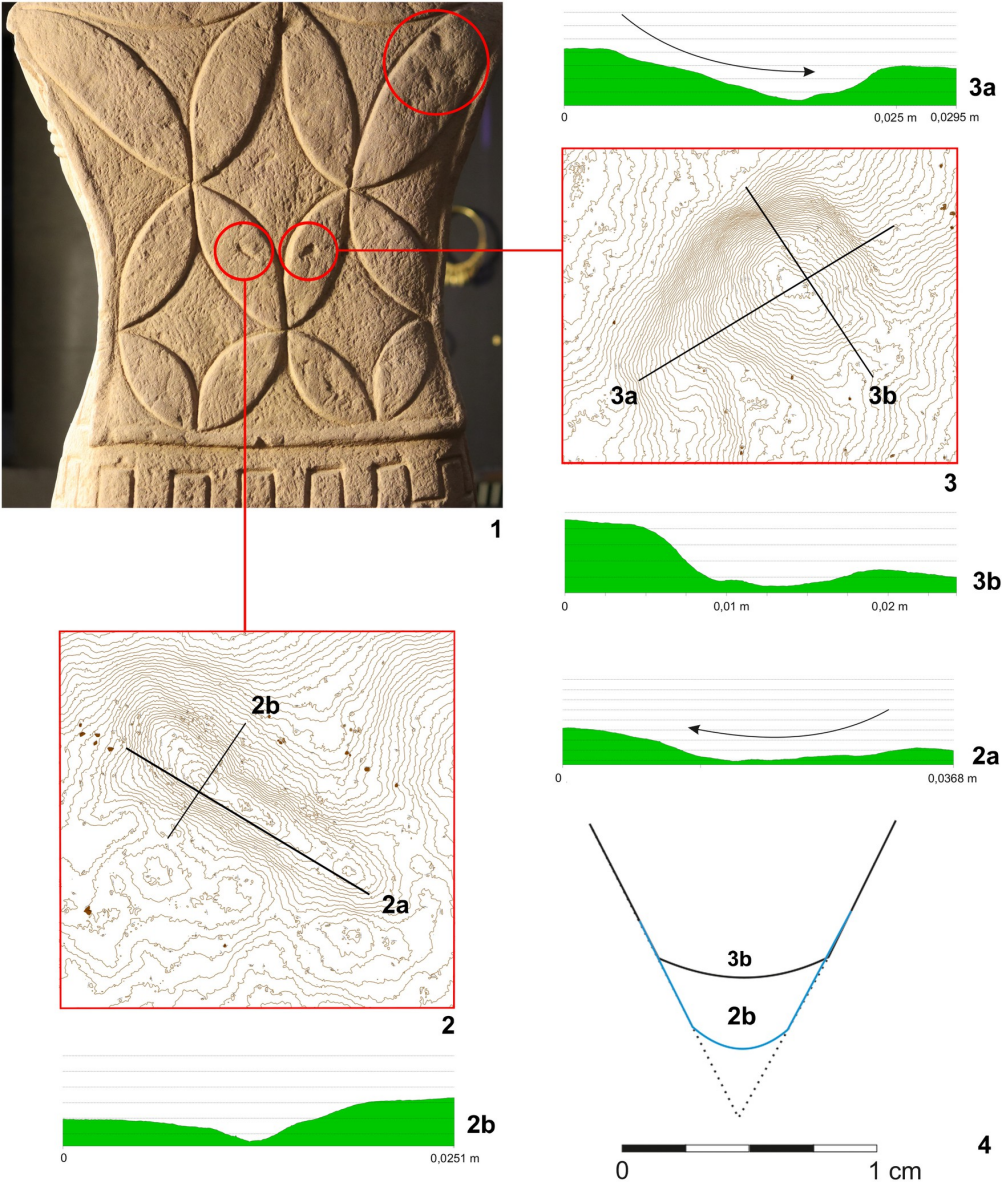
Statue 1 from Glauberg. 1 the sculpture´s back with the traces of the pick (in red circles); 2 isoline model of one of the traces; 2a longitudinal section of the trace; 2b transverse section of the trace; 3 isoline model of one of the traces; 3a longitudinal section of the trace; 3b transverse section of the trace; 4 reconstructed point of the pick as derived from the sections of the traces (author: M. Cihla and F. R. Václavík).

**Fig 17:**
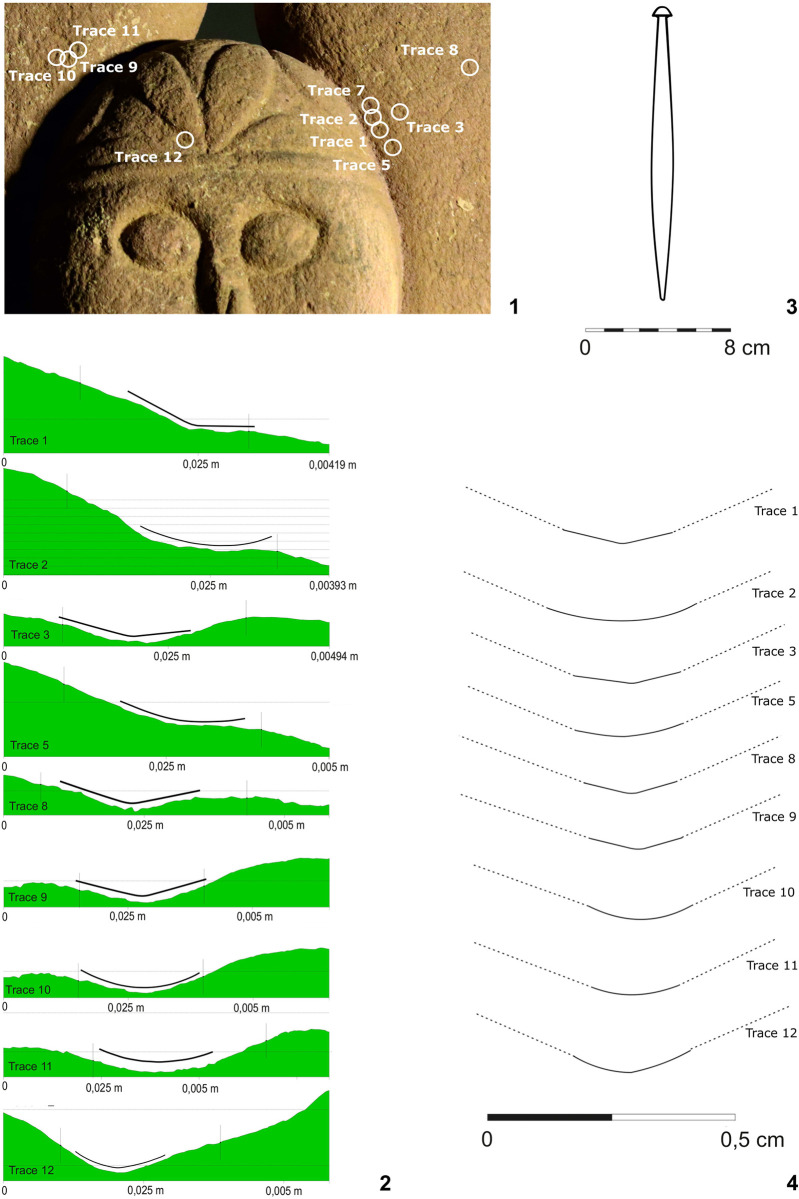
Statue 1 from Glauberg. 1 the traces identified in the head of the sculpture; 2 transverse sections of the selected traces on the head; 3 the reconstructed tool as derived from the identified traces; 4 the changing form of the blade of the tool indicating its sharpening (author: M. Cihla and F. R. Václavík).

**Fig 18:**
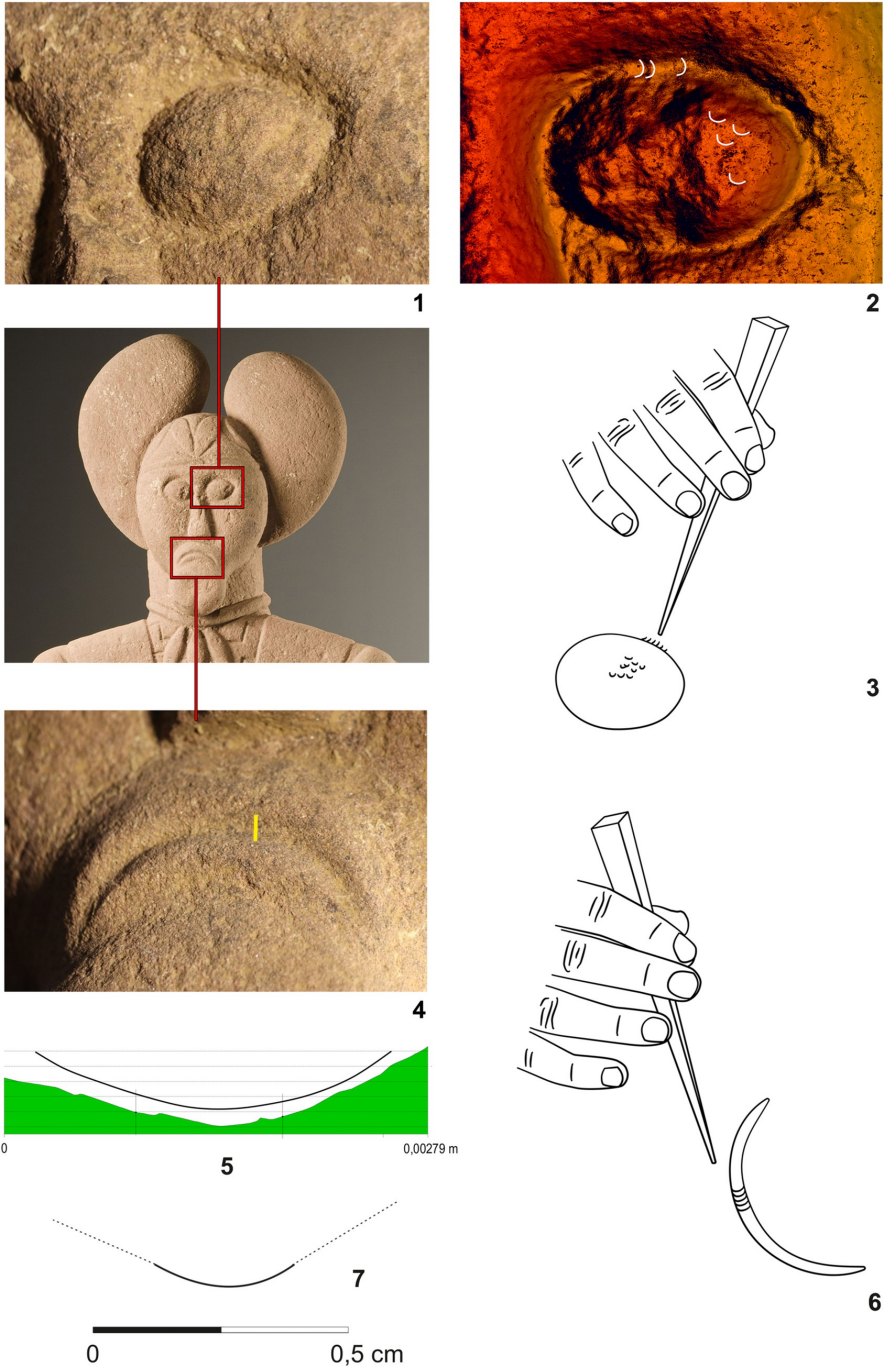
Statue 1 from Glauberg. 1 area of the eye; 2 digital model of the same area with indications of the arc-shaped traces; 3 reconstruction of the sculptor´s work with the relevant tool; 4 area of the mouth with the identified trace; 5 transverse section of the identified trace; 6 reconstruction of the sculptor´s work with the relevant tool; 7 reconstruction of the shape of the blade of the tool (author: M. Cihla and F. R. Václavík).

**Fig 19:**
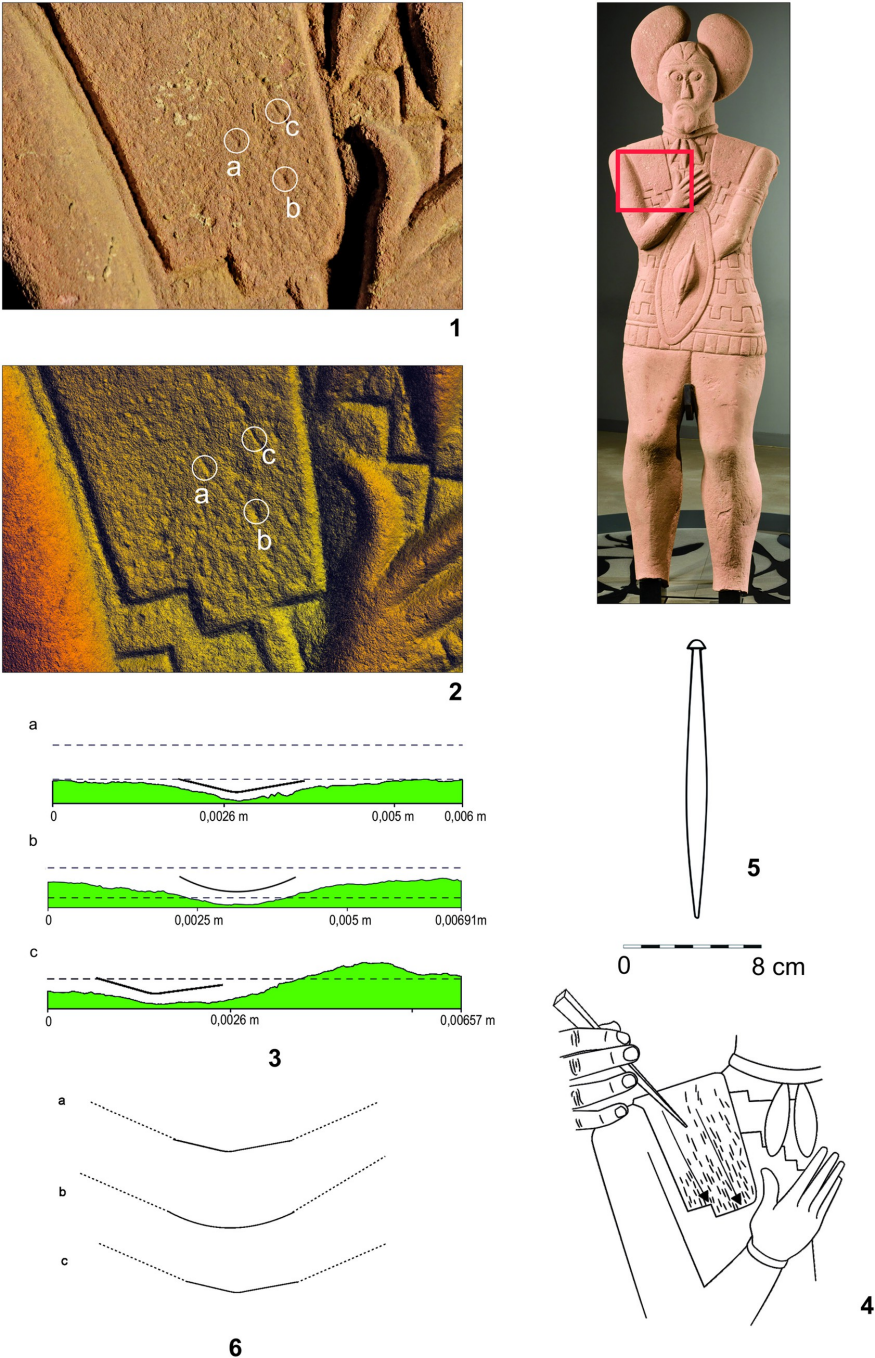
Statue 1 from Glauberg. 1 the traces identified above the left hand; 2 digital model of the same area with identified traces; 3 transverse sections of the identified traces; 4 reconstruction of the sculptor´s work with the relevant tool; 5 hypothetical reconstruction of the tool; 6 reconstruction of the blade of the tool as derived from individual traces (author: M. Cihla and F. R. Václavík).

**Fig 20:**
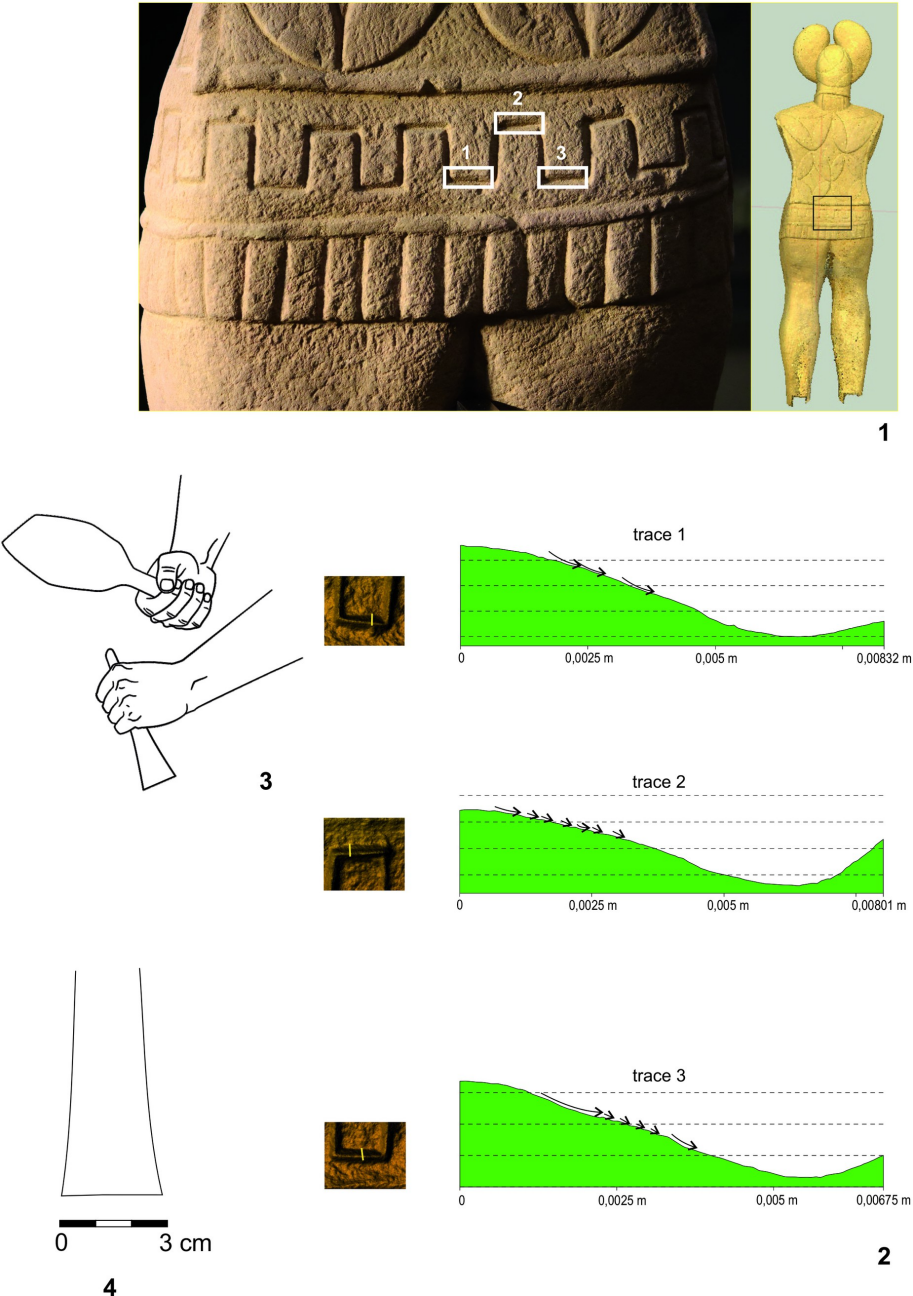
Statue 1 from Glauberg. 1 the traces of the broad chisel (1–3) identified in the meander line; 2 longitudinal sections of the traces 1–3; 3 hypothetical reconstruction of the sculptor´s work; 4 reconstruction of the shape of the blade of the tool (author: M. Cihla and F. R. Václavík).

**Fig 21:**
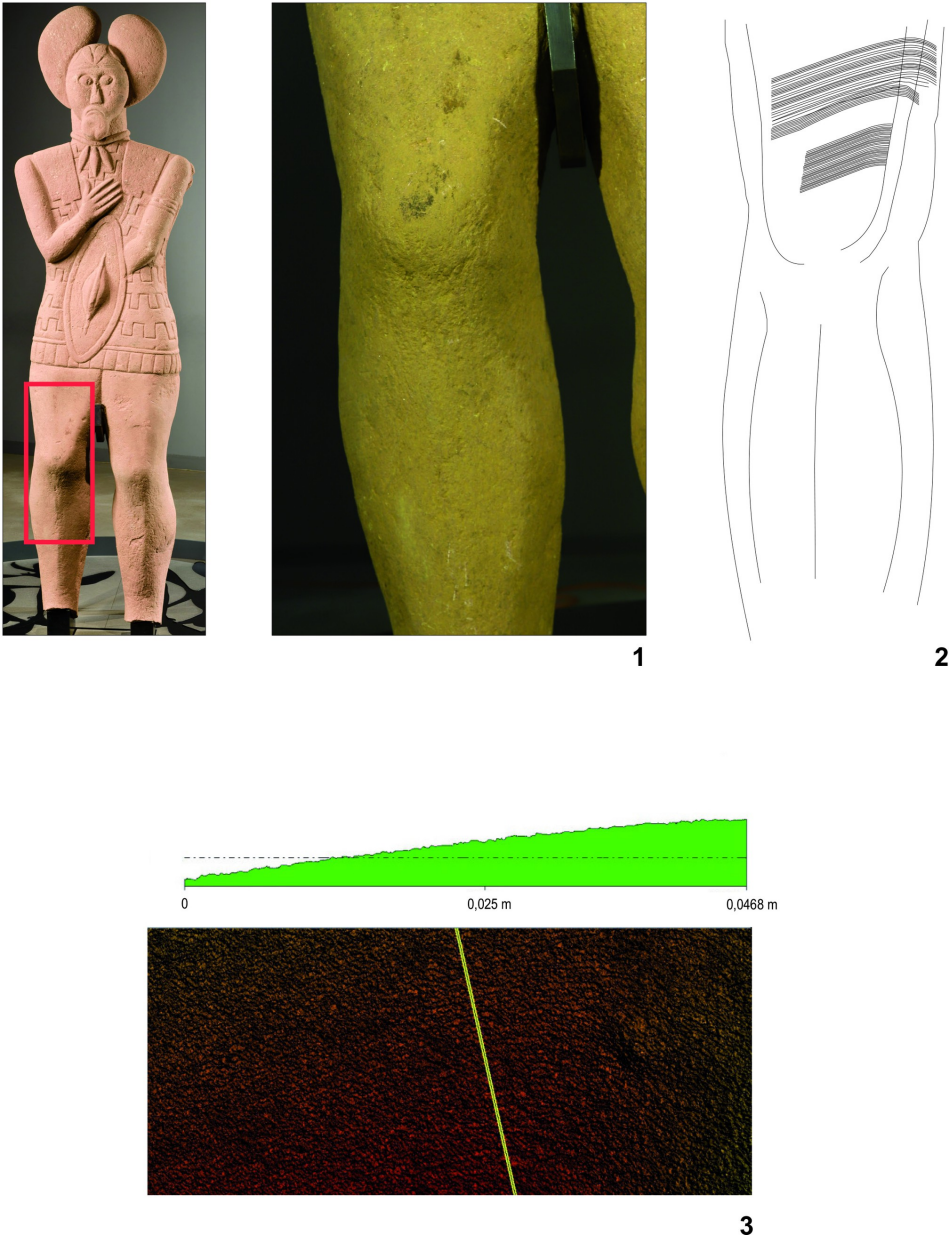
Statue 1 from Glauberg. 1 area of the thig with identified hair-thin parallel lines; 2 drawing of the documented situation; 3 the section of the area with the identified hair-thin parallel lines (author: M. Cihla and F. R. Václavík).

**Fig 22:**
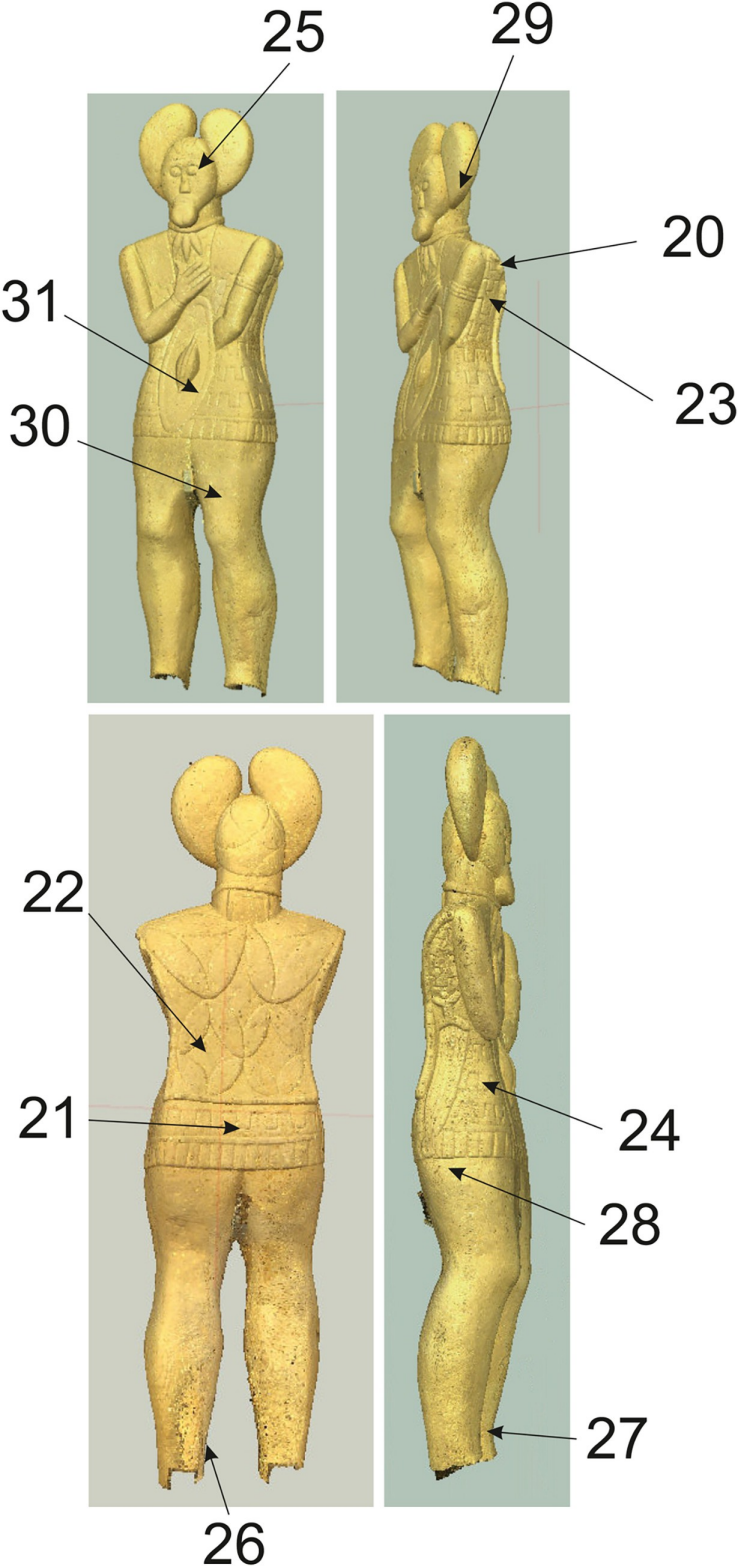
Points of the X-ray fluorescence measurements (author: M. Cihla and F. R. Václavík).

The well preserved statue 1 also yielded well preserved traces of particular tools. Firstly, the traces of the first phase of the sculpting of the figure were identified. These traces were found on many parts of the surface. The best-preserved traces were identified in the area of the sculpture´s back (Fig 15). These dynamic traces have a triangular form with a typical dot in the middle, representing a point of the strongest impact of the tool (Fig 15:1–2). They have a rounded shape in longitudinal section (Fig 16:2A–3A), which means that they were made by a **pick or double pick** with a handle. This tool was typical for rough working up of the surface (Fig 15:3).

Another identified tool is a slim **chisel** with a straight blade. The traces of this tool occur on all parts of the sculpture, because this tool was most likely the tool for finishing the sculpture´s surface, as was the case with the head fragment (Fig 9:4). The chisel´s traces are aligned behind each other in narrow parallel rows (Fig 19:2, 4). Except for the flat surfaces, this tool was used also for sculpting the details of the body (Figs 17 and 18) as well as of the fabric of the cuirass. The chisel´s blade is slightly rounded, and some traces show that it was occasionally sharpened (Fig 17:4). Although the blade must have been very thin (millimetres), the length of the tool is estimated at ca. 15–20 cm.

As mentioned above, the reconstructed chisel was the tool for the finishing treatment of the scuplture´s surface. Nevertheless, between the primary phase of the rough working up of the primary stone by the pick or double pick, and the finishing of the surface with the chisel, also an intermediate phase must be supposed, during which the bumpy surface left by the pick must have been levelled, probably using a chisel with a broader blade. Although the directly visible traces of this intermediate phase did not survive, occasionally we observed indications of broader cuts, subsequently being crossed at a right angle by traces of the forementioned slim chisel.

Another form of chisel was identified in the meander-shaped line on the cuirass. It was clearly a broad **chisel**, but we do not know its complete shape. Nevertheless, the traces indicate a blade around 3–5 cm wide (Fig 20:4). The individual cuts also suggest that the shape of the blade was gradually tapered, and the cutting edge of the blade was not sharp but slightly worn to a rounded form.

A very interesting phenomenon is represented by the completely polished thighs of the sculpture (Fig 21). In the microscopic image, it is apparent that this polishing consisted of thin and regular paralell lines. If this part of the thighs was polished in this manner, we suggest that for polishing harder stones were used, a technique which is often used by sculptors. However, in that case, the traces would be parallel, but not equally wide, according to the specific interior structure of the grinding stone. Nevertheless, the detected widths of the hair-thin grooves were all absolutely equal.

We experimentally verified, that such traces on the softer sandstone were produced by rubbing the surface in one direction with a sheep´s fleece. Thus, the explanation of this phenomenon could be that the stela, when still standing, was used by animals to rub against. Even though this interpretation may sound somewhat peculiar, it cannot be completely ruled out.

As for the XRF measurement (Fig 22:20–31; Table 2:ID 20–31), the results are unfortunately the least understandable, because there is practically no place on the surface of the sculpture where measurement of the interior of the stone is possible. We found only one such place, namely the measurement ID 20 (Fig 22:20; Table 2:ID 20). For this reason, the comparison of the values of the interior of the stone and its worked surface is only relative. One of the most interesting places is represented by the meander-shaped line on the lower part of the cuirass, made by a wide chisel. Two measurements (Table 2:ID 21, 24), both in the meander-shaped line, show an increase in the concentration of copper, whereas copper is completely absent in all the other measurements (with one exception: Table 2:ID 25). This could indicate that the broad chisel used for sculpting the meander line could have been produced from copper or a copper alloy.

The same may be said about the narrow chisel, used for the sculpting of the eye (Fig 18:1–3). Again, one measurement (Table 2:ID 25) had increased amounts of copper, in contrast to the other measurements, which did not detect copper.

Again, it is possible to cast doubts upon these assumptions due to hypothetic contamination by the surrounding soil (Table 3). However, it must be stressed, that from 12 measurements on the statue 1, copper was present only in two spots, one in the meander-shaped line and one in the eye. It therefore seems rather improbable, that the contamination would affect only the places worked by the tool and not the surrounding ones.

On other parts of the surfaces, some measurements (e.g. Table 2:ID 22–23, 26, 28, 31) showed slightly higher amounts of iron compared with the values from the interior of the stone (Table 2:ID 20). This might indicate a similar situation as in the case of the head fragment. We may thus suppose that the narrow chisel, used for the finishing of the greater part of the sculpture´s surface, was made of iron. However, also in this case the assumption on the material would be rather hypothetic, due to a possible contamination by the surrounding soil.

## 4. The identified tools and the archaeological reality

In conclusion, concerning the tools identified on the fragments of the head and leg, and on the complete statue, we could recognize:

- **a narrow chisel,** probably made of iron (fragment of the head; Figs 8 and 9)
- **an adze,** hypotheticaly made of bronze (fragment of the leg; Figs 11–14)
- **a pick or double pick,** made of an unknown material (Figs 15 and 16)
- **a narrow chisel**, hypothetically made of iron (Fig 17)
- **a narrow chisel,** probably made of bronze (Figs 18 and 19) and
- **a wide chisel** (Fig 20), probably made of bronze (statue 1)

To understand the tools better, it is necessary to compare the identified types with existing tools found in contemporary Iron Age archaeological contexts. It is also desirable to emphasize one problem related to the terminology. The classification of the tools mentioned above as “chisels” or “adzes” is rather subjective. The difference between these two tools is, that the blade of the adze is oriented transversely, furthermore the adze is a tool set in a haft. The chisel is a hand tool, in which the working blade may be used both transversely or parallel. One might suppose that the socketed tools are automatically adzes, since it may be considered that the socket served for attaching a haft. However, using only a short wooden shaft fixed in the socket, the socketed tools may well have been used also as chisels.

**Fig 23:**
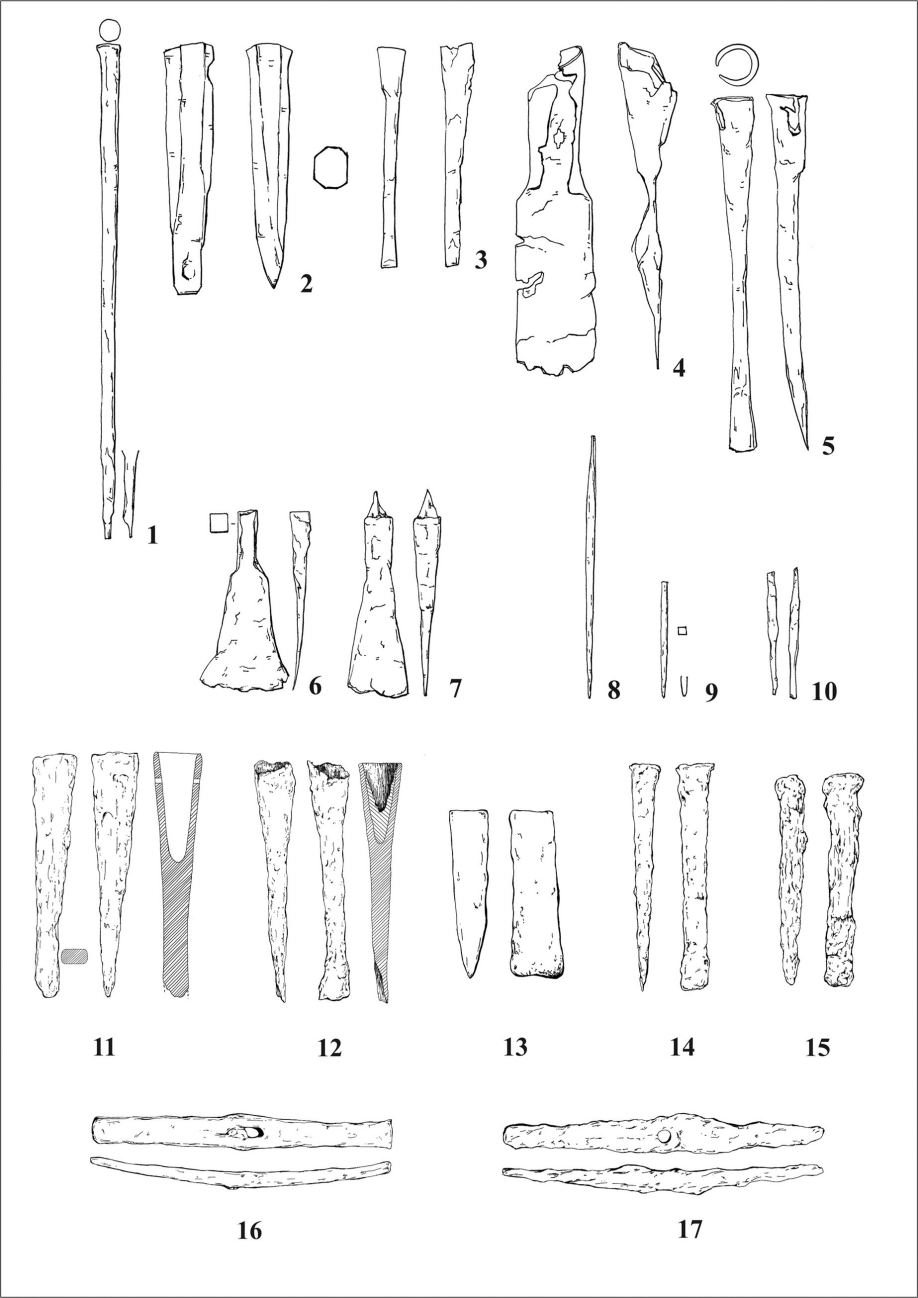
Finds of the corresponding tools from Germany and France: 1 Manching (after [100], Nr. 82); 2 Bibracte (after [99], Nr. 43); 3 Glauberg (after [105], Abb. 72); 4 Bibracte (after [99], Nr. 93); 5 Dünsberg (after [106], Nr. 18); 6 Manching (after [100], Nr. 103); 7 Bibracte (after [99], Nr. 64); 8 Manching (after [100], Nr. 238); 9 Bibracte (after [99], Nr. 54); 10 Nidderau (photo A. Ulbrich); 11–17 Heuneburg (after [102], Nr. 1840–1841, 1849–1851, 1856–1857 (drawing: A. Musilová).

Because fine examples of stone masonry of the Early La Tène period is not widely known in the Central European context, it is may be supposed that there are also few proper stonemason´s tools from this period. It is likely that the tools for sculpting the Glauberg statues were, in fact, tools normally used for working with wood or metal. The only tools which may have been made specifically for working with stone are very large chisels (up to 55 cm in length) with a massive shaft (Fig 23:1–2), like that ones from Bibracte (dép. Saône-et-Loire) [99], Manching (Ldkr. Pfaffenhofen a. d. Ilm) [100] or one specimen from the hoard of Kelheim (Ldkr. Kelheim) [101]. Furthermore, a large quantity of tools were found in the Late La Tène Oppida like Manching or Bibracte, which are significantly later than the Glauberg statue. However, tools sometimes have the tendency not to change their shapes over time–as long as the way they were used did not change, there was no need to change the tool itself, so they might look very similar over a longer period of a couple of hundred years.

Several tools that correspond very well with our reconstructions have been found in Heuneburg. Two tools classified as “Tüllenmeissel” [102] are identical with our reconstructed spout chisels or adzes (Fig 21:11, 12). Some tools (Fig 21:13–15) might have been also used as broad chisels [102]. Also hammers [102] could have been used as adzes (Fig 21:16, 17), similarly as below mentioned finds from Libčice/Chýnov. Leaving aside the problems with stratification of some Heuneburg finds, presented tools may be dated back to the late 7^th^ unitil the early 5^th^ century BC, what corresponds with the general chronology of the settlement in Heuneburg [103]. A relevant site is in this respect also the hillfort Kleiner Gleichberg in Thuringia, where tools dated back to the Late Hallstatt and Early La Tène period have been uncovered [104].

To start at the Glauberg itself, there are fortunately some iron tools which were found on the Glauberg Plateau by H. Richter (Fig 23:3). Two iron socketed chisels („Tüllenmeißel“) were probably found in 1935 in ditch C on the northern border of the Plateau in a layer that has been dated to the Early La Tène period by Richter [105]. Richter’s chronological observations are not always correct, so they could potentially also date to later periods. The shape of the working edge of these tools is very similar to the edge of the reconstructed tool used for sculpting the fragmentary isolated leg (Fig 12: 1, 3 and 13:2, 4). Unfortunately, they are not preserved today, so further examinations are impossible. In an unpublished manuscript, Richter suggested that they were used to mine stones, but according to H. Baitinger [105] they were mainly used for working with wood. If these tools were used hafted, with the working edge orientated transversally, they would have left traces on the stone surface identical to those which are today visible on the isolated leg (Figs 11:1 and 12:1–4 and 13:1–3 and 14:1–2).

In Bibracte, there was found a socketed iron tool (Fig 23:4), which seems to be too big for a chisel; instead, it could be an adze [99]. Because the wooden haft of the tool is not preserved, both options could be possible.

Socketed chisels of the same type were used until the Late La Tène period, as the tools from Manching demonstrate. Jacobi describes these tools, ca. 22 cm long and with a short wooden shaft, which were supposedly used for cutting mortise joints [100]. This kind of tool (Fig 23:5) is also known from the Lt C/D Dünsberg oppidum (Lkr. Gießen) [106], the Lt C/D Heidetränk oppidum (Hochtaunuskreis) [107] and the Steinsburg oppidum (Lkr. Hildburghausen) [108], where the most important settlement periods are in Ha D/Lt A and Lt C2/D. This tool is similar to the two socketed chisels from the Glauberg, and are comparable with the reconstructed tool, used for sculpting the isolated leg from this site.

Of special interest, apart from settlement finds, are hoards which were presumably deposited by craftsmen, because they can contain a wide variety of tools. Several chisels of different kinds originate from two smith´s deposits from Langenfeld (Lkr. Hameln-Pyrmont) [109] and the Heidelberg (near Schweinthal, Lkr. Forchheim) [110], together with hammers, files, hearth shovels, spearheads, fire dogs and workpieces. Though the latter depot was dated to the Early La Tène period by H.-U. Abels, it could also be dated later in the La Tène period, as the origin of the hoard is not documented, and the finds themselves cannot be precisely dated within the Iron Age.

Most of the chisels from Manching and other Late La Tène locations do not have such a broad blade like the reconstructed tool which was used to create the cuirass of statue 1 (Fig 20). Only one specimen [100] could be considered as suitable (Fig 23:6). From Manching, we know–beside the iron specimen–also bronze flat chisels, but these have narrow blades [111]. The blades up to 4 cm in case of the wood working tools („Tüllenbeitel“) have been found at Bibracte (Fig 23:7) [99] and Manching [100]. They are known since the Hallstatt period [99]. A hoard from the end of the Early La Tène period was found on the southwest slope of the Schlossberg near Neuenbürg (Enzkreis), with three scythes, one flat chisel and one gouge [101]. In the opinion of J. Röder, flat chisels were first used in the Hellenistic period, but his suggestion is no longer valid [94], as it has been shown that they were used since the Hallstatt period [99].

Narrow chisels are very similar to the pin punches („Durchschläge“) known from Bibracte or Manching. Their shape corresponds to the reconstructed tool which was used on the head of statue 1 and for the isolated head (Figs 8 and 9, 12–14). In Manching they are up to 10 cm (cf. Fig 23:8), in Bibracte up to 15 cm (cf. Fig 23:9) long, with a square or round cross-section in the middle, and a narrow rounded working blade. Typical for the La Tène specimen is the tapered shape [99]. Usually, they were used to punch holes in hot or cold metal. According to D. Mölders, the large specimens could also be used as chisels for stonework [99]. Pin punches were found in Lt C/D contexts, but G. Jacobi assumes, considering the traces on Late Hallstatt and Early La Tène objects, that these tools were already used in these periods [100]. In a Lt B2 warrior grave in Nidderau (Ldkr. Main-Kinzig-Kreis), only 15 km from the Glauberg, two iron tools were found (Fig 23:10), whose shape is quite similar to the described tools (length: 9,6 cm and 10.5 cm). They have a round edge on one side and a square edge on the other side, and are thickened in the middle [112]. Graves with tools in the Early La Tène period are uncommon, but we know other finds from a Lt B grave with weapons (sword, lance) and smith’s tools (hammer, anvil, two chisels) in grave 13 from Au am Leithagebirge (Kr. Bruck an der Leitha, Lower Austria) „Hutweide”[113, 114].

As for the double pick for the rough work, two iron double pick hammers, 24 cm in length, are known from Mayen (Lkr. Mayen-Koblenz), which is famous for its Pre-Roman and Roman basalt mines. According to the tool marks on La Tène Quern-Stones („Napoleonshüte“), they were used since the Early La Tène period [115].

**Fig 24:**
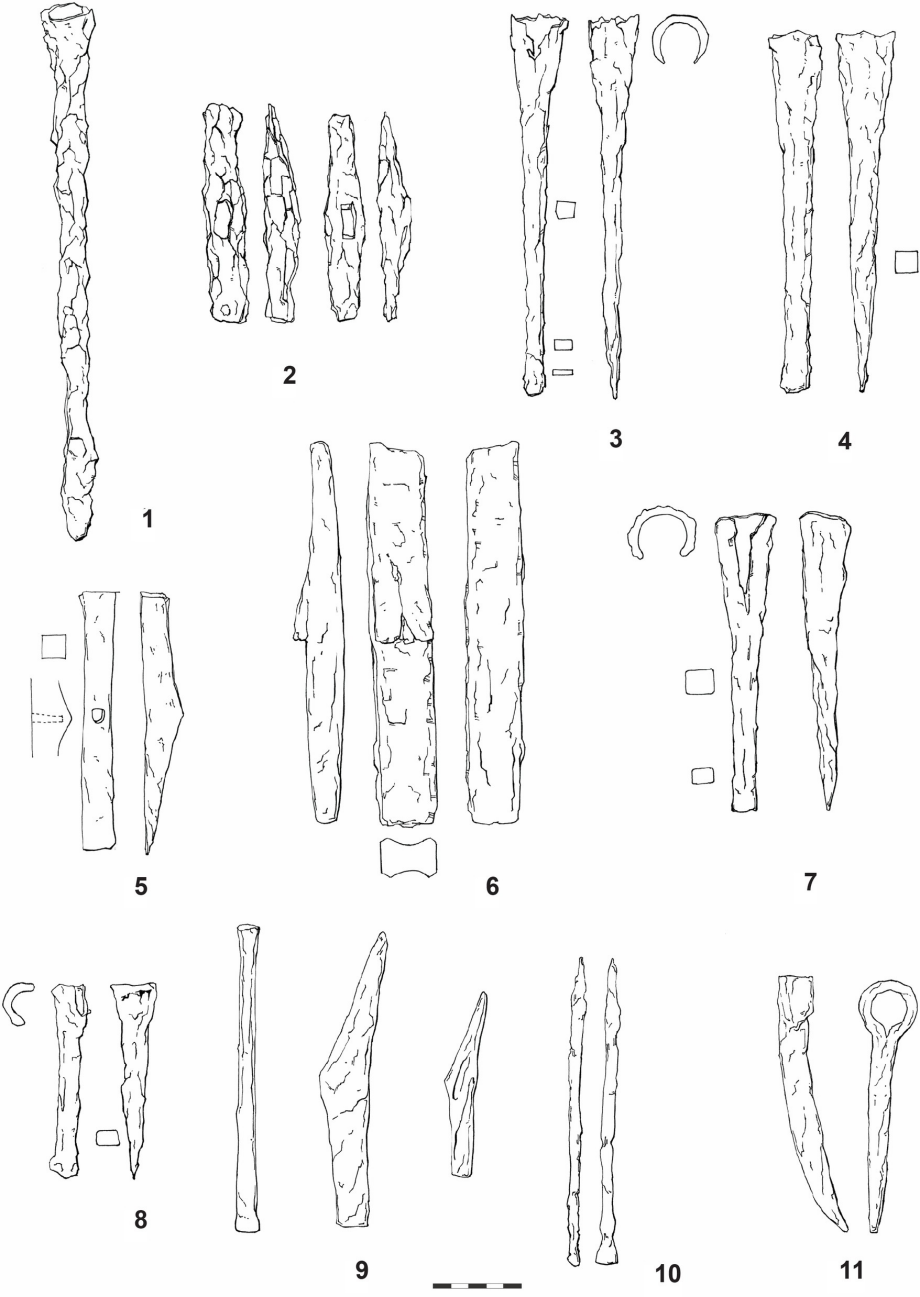
Finds of the corresponding tools from Bohemia: 1–2 Libčice nad Vltavou-Chýnov (after [121], 243); 3–6 Kolín (after [117], Abb. 13:1, 17:1–2, 18: 2); 7–8 České Lhotice (after [119], obr. 72:3, 6); 9 Stradonice (after [120], obr. 204:1–3); 10 Řehnice (after [121], 437); 11 Bezdědovice (after [121], 150–151) (drawing: A. Musilová).

The Bohemian basin–like more or less every region in Europe–is not an area with a frequent occurence of Celtic stone sculpture. Nevertheless, this area should not be omitted in a search for the appropriate comparisons to the relevant tools, since it is an integral territory of the La Tène *oecumene*. Moreover, if we suggest that some tools were meant for working wood or for making quern-stones, we should be able to identify comparable finds here too. For example, the iron socketed chisel (Fig 24:1) from Libčice nad Vltavou (okr. Praha-západ) in Central Bohemia [116] is a suitable comparison to the adze, also in the width of the blade, which served for the working up the isolated knee (Fig 13:5). Also, the hammer from the same site (Fig 24:2) [116] could have been used in the same way (in case of a blade that was originally sharp; nevertheless, its original form can be only estimated due to corrosion; this problem concerns also the two below mentioned hammers). While both tools are dated to the Late Hallstatt/Early La Tène period, two iron socketed chisels in a hoard from Kolín (okr. Kolín) in Central Bohemia (Fig 24:3–4) date to the Late La Tène period [117, 118]. The widths of their blades are again comparable with the width of the reconstructed tool. The Kolín hoard also included a hammer (Fig 24: 5) [117], which–as in the case of the Libčice hammer–could have been used as an adze. The same may be said for the third chisel from this hoard (Fig 24:6) [117].

Socketed chisels with a blade width of ca. 1,7 cm, are also known from the Late La Tène oppidum České Lhotice (okr. Chrudim) in Eastern Bohemia (Fig 24:7–8) [119]. The oppidum of Stradonice (okr. Beroun) in Central Bohemia yielded one chisel and two hammer-like tools (Fig 24:9), which–hypothetically—could also have been used as adzes [120].

Several tools, which deserve mention here, are deposited in the collections of the National Museum in Prague. Two bronze narrow chisels were found in Lžovice (okr. Kolín) in Central Bohemia, allegedly in a hoard (inv. no. 16247–16248). They were dated to the transition between the Late Bronze Age and Early Iron Age. Further tools date to the Late La Tène period: two narrow bronze chisels, whose blades are less than 1 cm wide, from Stradonice (inv. no. 65124, 81462), one iron socketed chisel from Tetín (okr. Beroun; inv. no. 45654), and two iron narrow chisels from the oppidum of Třísov (okr. Český Krumlov) in Southern Bohemia (inv. no. 224401, 252934). While these narrow chisels may serve as general analogies (some of them also in the width of the blade) to the reconstructed tools used for working up the surface of the complete statue 1 and the isolated head (Figs 17–19, 8–9), the Tetín socketed chisel corresponds to the adze used for sculpting the leg of statue 2 (Figs 11–14).

One narrow chisel (Fig 24:10), generally corresponding to the narrow chisels used in the manufacture of the Glauberg statues, was found during the excavation of a sunken hut and adjacent silo in Řehnice (okr. Mladá Boleslav) in Central Bohemia [121]. The context was dated to the 3rd–2nd century BC, thus approximately to the Middle La Tène period.

The Late La Tène hoard from Bezdědovice (okr. Strakonice) in Southern Bohemia [121, 122] included at least 47 intact and 137 fragments of various tools. It included a single pick–with a socket for the haft and a single point (Fig 24:11). This tool is comparable with the pick or double pick, used during the sculpting of the basic form of statue 1.

Similarly, as in case of the German analogies, the majority of the tools presented here date to the Late La Tène period. Nevertheless, the case of the Libčice find clearly shows that the socketed chisels, frequently occured in the Late La Tène period, were already known during the 5th century BC. This is also the case with the “hammer-like” hafted tools, which could hypothetically have been used as adzes. The Lžovice narrow chisels indicate that these tools were known already before the beginning of the Early Iron Age.

**Fig 25:**
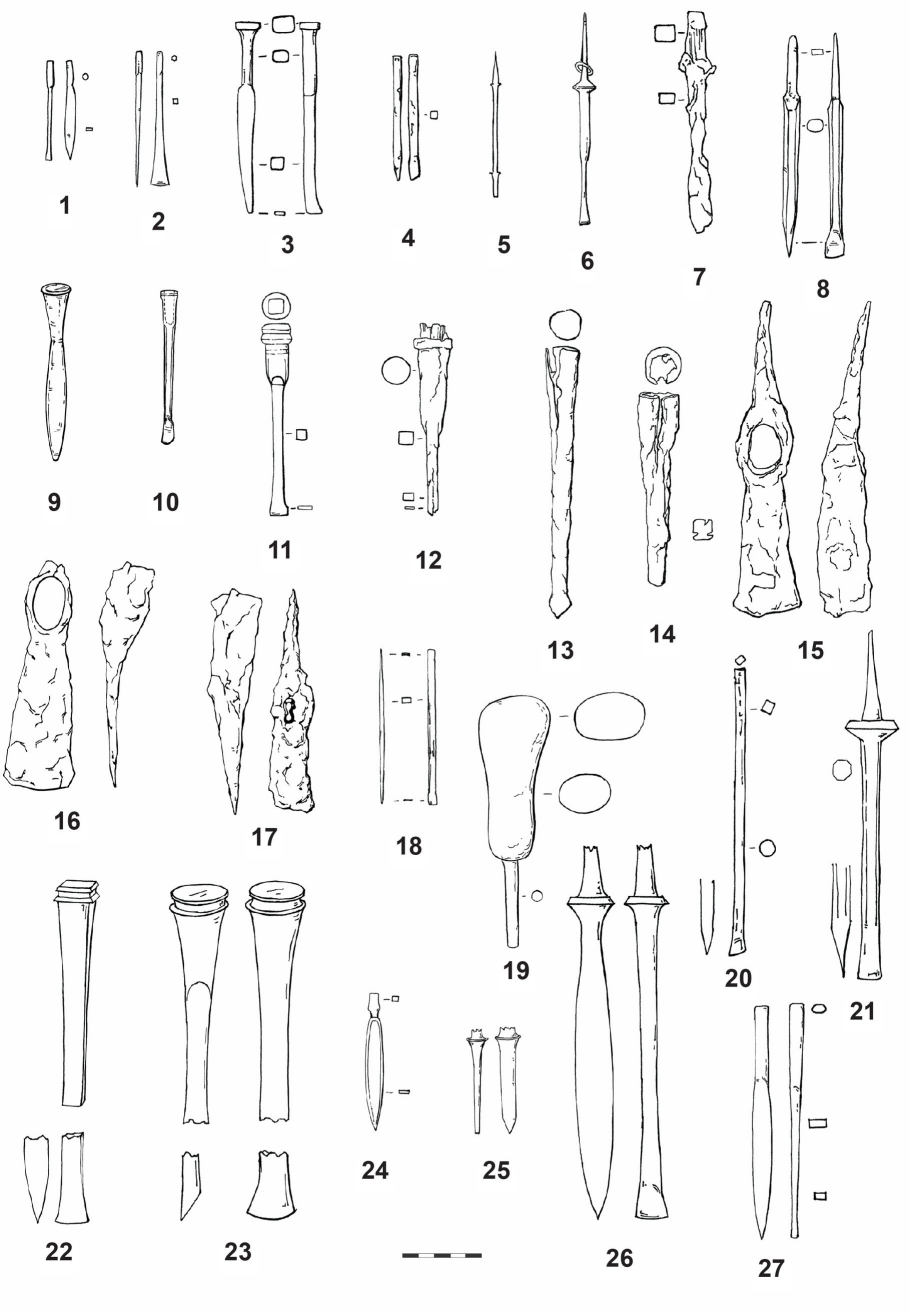
Finds of the corresponding tools from Italy: 1–2 San Vito al Tagliamento; 3, 8 Veio; 4, 7, 15 Tursi; 5 Cerveteri; 6, 9 Vetulonia; 10 Vulci; 11–12, 16 Pontecagnano; 13 Roggiano Gravina; 14 Pitecusa; 17 Tolentino S. Egidio; 18 Castione Marchesi; 19 Monte Titano; 20 Seconda Torre; 21–26 Bologna; 27 Monte Cavanero (after [123], Figs 3 and 4; [125], Figs 13 and 14) (drawing: A. Musilová).

As mentioned in the introduction, the area of the Apennine penisula is of a substantial significance for the diffusion of knowledge of monumental stone sculpture to the transalpine area. Thus, it seems not to be a coincidence that we find many analogous tools during the Early Iron Age also here [123]. As for the socketed chisels, particular analogies to the above-mentioned tools are represented by finds from Vetulonia (prov. Grossetto), Vulci (prov. Viterbo), Pontecagnano (prov. Salerno), Roggiano Gravina (prov. Cosenza) and Pithekoussai (Ischia, prov. Napoli) (Fig 25:9–14) [123]. C. Iaia quotes the opinion of Jacobi [100] and Mayer [124], that these tools were intended for working with wood, and he stresses the fact that the iron socketed chisels were also distributed in southern Italy; Iaia classified the chisels according to the size of the blade [123].

Very good examples of chisels are also known from Italy. These are the finds from San Vito al Tagliamento (prov. Udine), Veio (prov. Roma), Tursi (prov. Matera), Cerveteri (prov. Roma) or Vetulonia (Fig 25:1–8) [123]. All such tools may well have been used for working up or polishing the surface of the isolated head or individual parts of the complete statue from the Glauberg.

Suitable examples of chisels and socketed chisels are also well represented among the Italian tools which are supposed to have been used in metallurgy [125]. Chisels, such as the finds from Monte Cavanero (prov. Cuneo), Castione Marchesi (prov. Parma), Monte Titano (San Marino) or Bologna-S. Francesco (prov. Bologna) (Fig 25:27, 18, 19, 20, 24–25) [125] are comparable with the chisels reconstructed from the traces on the isolated head or on statue 1 (Figs 8 and 9 and 17–19). However, the Italian finds also include examples with a wider blade, such as the chisels from Bologna-S. Francesco (Fig 25:21, 26) [125]. Similar chisels could have been used for creating the meanders on the cuirass of the complete statue (Fig 20). Finally, the North Italian finds include socketed chisels, again in the famous hoard from Bologna-S. Francesco (Fig 25:22–23) [125], which could also have been used, fixed to a haft, as adzes. It is possible that similar tools were used for working the surface of the leg of statue 2 from the Glauberg (Figs 11–13).

Also the iron “axes” with transverse blade, which might have been used as adzes, are well represented in the Apennine peninsula. These include the finds from Pontecagnano or Tolentino-S. Egidio (prov. Macerata) (Fig 25:16–17) [123]. Similar tools could well have been used for working up the surface of the knee of statue 2 from the Glauberg (Fig 11–14).

One “axe” from Tursi (Fig 25:15) [123] has one side flat and another side pointed. A similar instrument could have been used for the rough working during the primary phase in the manufacture of statue 1 from the Glauberg (Figs 15 and 16).

The majority of the Italian tools mentioned here are interpreted as tools for working with wood, metal or generally soft materials [123]. Nevertheless, without doubt they may also have been used for stonemasonry. This is confirmed directly by the traceological research of statue 1 and the related fragments, and it is also admitted by Iaia for the more massive types of chisels [125]. The use of tools specifically for working with stone is genrally only conceived in the case of very large and massive examples. However, the bronze adze used for detaching limestone blocks in the quarry of Cava Magi near Tarquinia, as reconstructed from the traceological marks and verified by experiment, was only 13 cm long.

## 5. Analysis of the Glauberg fragments and the research of F. Bodis and T. Schlick

The year 2018 saw the new publication by Udo Recker and Vera Rupp [126], focussed on many interdisciplinary analyses of various materials from the excavation of the three rich Glauberg graves, and the statues found next to them. One chapterof Frank Bodis and Thilo Schlick [127], is dedicated to the sculpting of the copy of the complete Glauberg statue by modern means, and using modern tools. This unique work offers the possibility of proper comparison of our observations and conclusions with the sculpting procedure described by the above-mentioned authors.

### First phase

The sculpting of the prefabricate is similar in the case of the modern sculptors as in the case of the ancient sculpture. Modern sculptors mainly used the handled pick or the smaller handled double pick for creating the rough shape of the figure, and they also used crandall hammer, a tool with a spiked blade. The sculptor of the Early Iron Age also used a pick, whereas the crandall hammer was identified, since it is probably a modern invention.

The sculptor of statue 1 (and most likely of the other, fragmented statues as well) worked up the rough shape with the figure lying in a horizontal position. The blows of the pick do not have just one direction, but were made from many different directions, indicating that the sculptor frequently changed his position.

### Second phase

In the second phase of work, the contemporary sculptor used a claw chisel and sometimes also a hand pick. Nevertheless, we must emphasize that there were no signs of the use of a claw chisel on the Glauberg complete statue and fragments; however, traces of the use of claw chisels have been found on stone sculptures from Ancient Egypt, Etruria and Greece.

The tool used by the Early Iron Age sculptor during the second phase of sculpting is unknown. However, we suspect that the tool of the second phase could be indicated by the fragment of the leg of statue 2, with traces of the handled adze. It is very probable that such a tool was also used in the case of statue 1 and the fragment of the head, although these traces are not apparent anymore, due to finishing the surface during the third phase.

### Third phase

In the third phase of work, the Early Iron Age sculptor used a narrow and long hand chisel with a rounded blade, which was repeatedly sharpened in the form of a lancet arch and had a blunt point. The sculptor worked in parallel rows, and it this stage of work was probably very time-consuming. This kind of tool, used in the same way, has also been demonstrated for the sandstone head from Závist near Prague [32]. In the third phase of work, the modern sculptor used various chisels with wider blades.

## 6. Conclusion

In the context of his research on the Hirschlanden warrior, J. Röder [94] (cf. supra) already mentioned the possibility of observing the “fingerprints” of two different sculptors. The same question is also legitimate in our case. Indeed, it is very tempting to suppose two different ways of the work, and two different sculptors, in the treatment of statue 1 and the fragment of the head on the one hand, and the fragment of the leg on the other hand. The assumption of two “fingerprints” would be highly likey, if we could be sure that the contemporary state of the leg corresponds with the finished look. This seems highly likely, but nevertheless, as this is not absolutely certain, the participation of more than one sculptor remains debatable.

The initial thoughts on the Hirschlanden warrior also included the hypothesis that a sculptor of Mediterranean origin could have participated in the process of its manufacture. But today, this idea seems highly improbable. The same may be said about the idea that the hypothetical Mediterranean sculptor created the Hirschlanden warrior in a “Celtic manner”, as contemplated by Röder. Both hypotheses seem just as improbable for the statue from the Glauberg.

**Fig 26:**
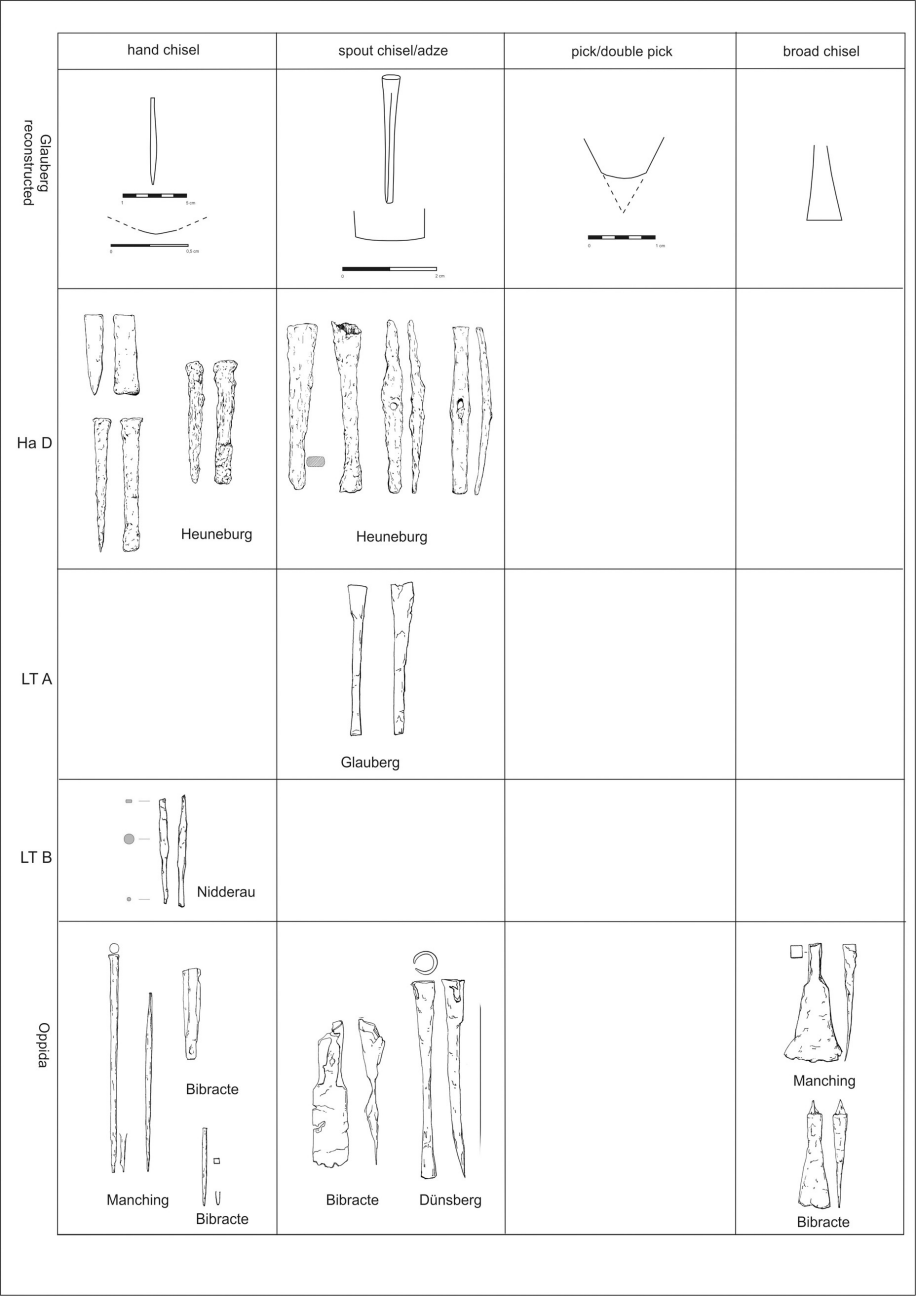
A comparative table of the reconstructed shapes of tools used during the sculpting of Glauberg fragments and the examples of tools from Germany and France (individual finds are not depicted in the same scale) (author: M. Trefný and A. Musilová).

**Fig 27:**
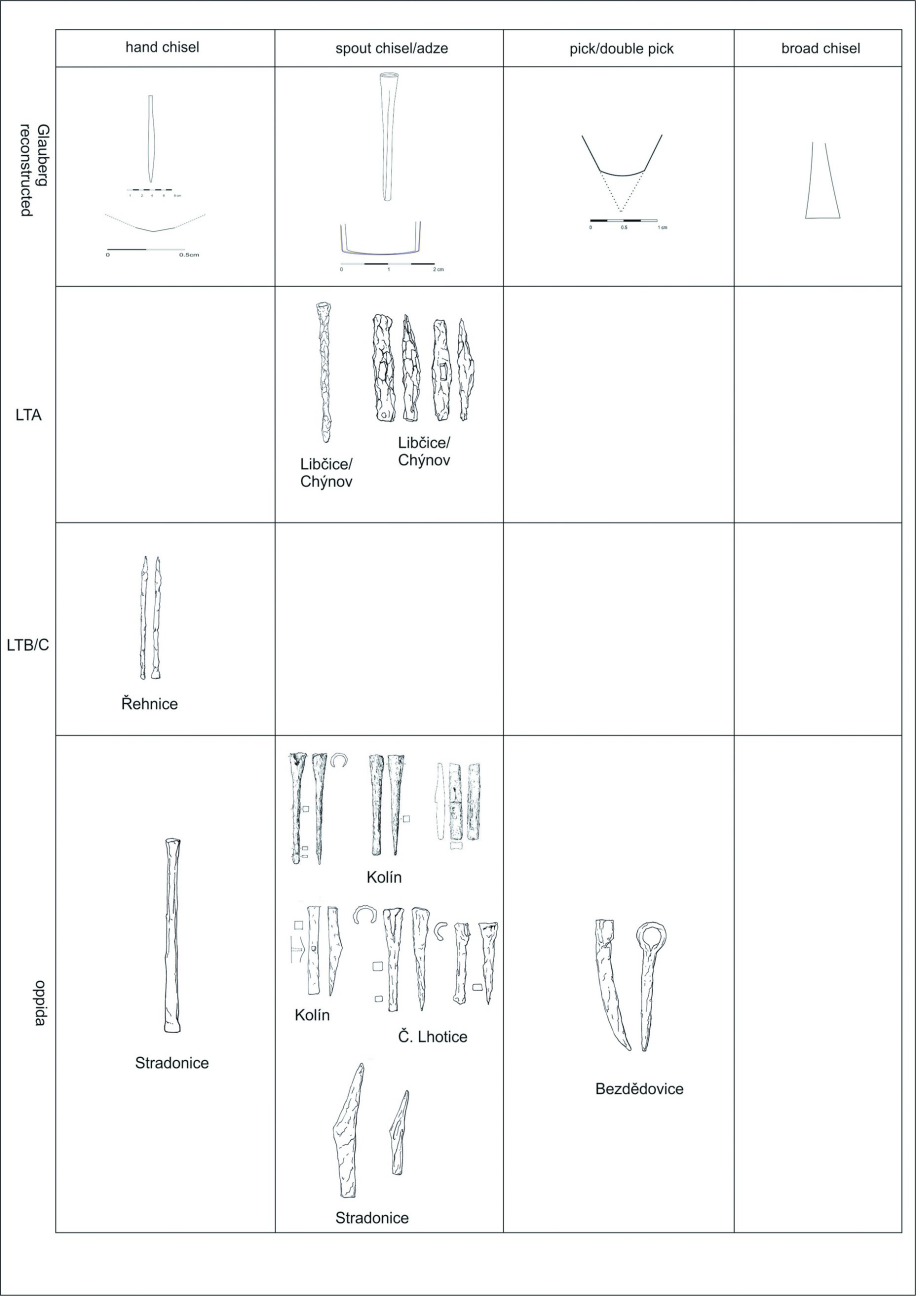
A comparative table of the reconstructed shapes of tools used during the sculpting of Glauberg fragments and the examples of tools from Bohemia (individual finds are not depicted in the same scale) (author: M. Trefný and A. Musilová).

**Fig 28:**
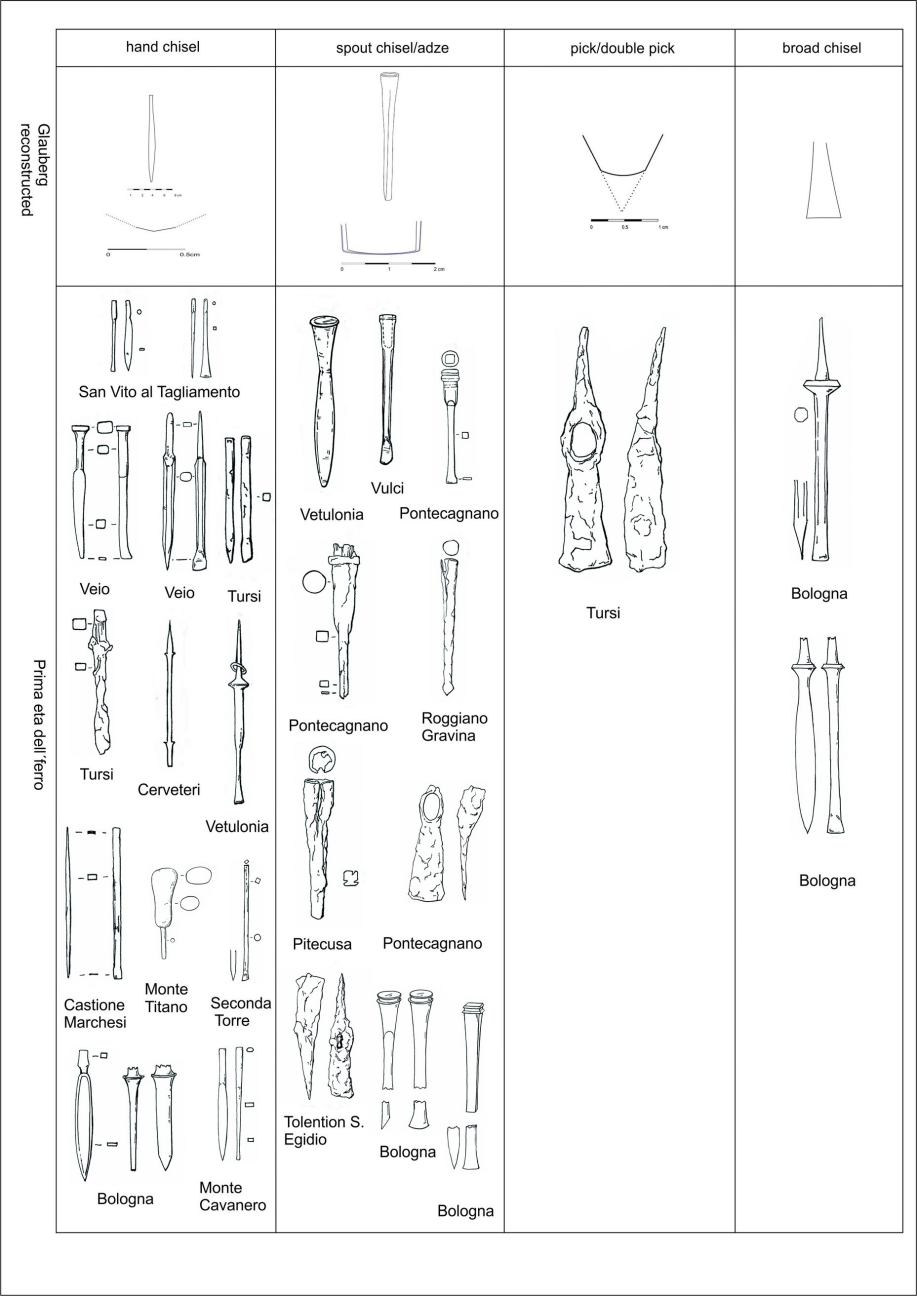
A comparative table of the reconstructed shapes of tools used during the sculpting of Glauberg fragments and the examples of tools from Italy (individual finds are not depicted in the same scale) (author: M. Trefný and A. Musilová).

**Fig 29:**
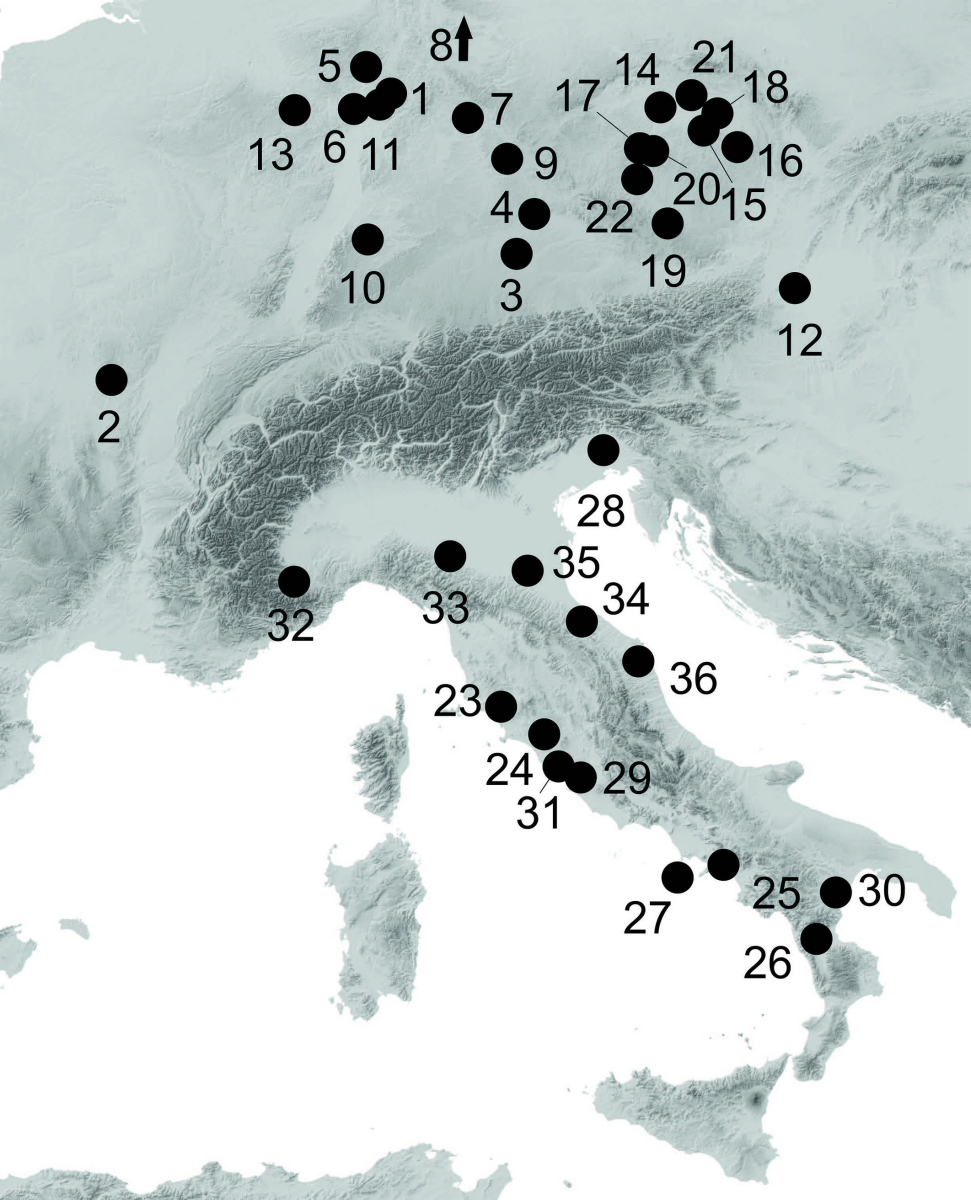
Sites with the comparable finds of tools mentioned in text. 1 Glauberg, 2 Bibracte, 3 Manching, 4 Kelheim, 5 Dünsberg, 6 Heidetränk, 7 Steinsburg, 8 Langenfeld, 9 Heidelberg, 10 Schlossberg, 11 Nidderau, 12 Au am Leithagebirge, 13 Mayen, 14 Libčice nad Vltavou, 15 Kolín, 16 České Lhotice, 17 Stradonice, 18 Lžovice, 19 Třísov, 20 Tetín, 21 Řehnice, 22 Bezdědovice, 23 Vetulonia, 24 Vulci, 25 Pontecagnano, 26 Roggiano Gravina, 27 Pitecusa, 28 San Vito al Tagliamento, 29 Veio, 30 Tursi, 31 Cerveteri, 32 Monte Cavanero, 33 Castione Marchesi, 34 Monte Titano, 35 Bologna-S. Francesco, 36 Tolentino-S. Egidio (author: M. Trefný).

The stonemason´s tools, as reconstructed from the working traces on the Glauberg sculptures, correspond quite well with real tools, as represented by archaeological finds (Figs 26–29). The double pick hammer was used at least since the Early La Tène period, as shown by the example from Mayen. Narrow chisels or pin punches made of iron are also represented in the archaeological record, and the same is true for bronze examples. The edges of flat chisels are usually not so wide as we reconstructed from the traces on the cuirass of the statue, although some examples with wider blade do occasionally occur in archaeological contexts. The tool marks which we assigned to an adze could also be from a socketed chisel, used in the manner of an adze, like the ones from the Glauberg itself, which have frequently been found in both contemporary and later contexts. Most of these were made of iron, but bronze examples were also used, as the Italian finds indicate. On the other hand, we know a flat socketed adze from Bibracte, which is made of bronze. Most of the comparisons were found in Middle or Late La Tène settlements, like Bibracte, Manching, Dünsberg or the Heidetränk Oppidum, but we assume that they were also used in earlier phases.

The traces identified on the surface of the complete statue, as well as on the fragments of the leg (statue 2) and the head (statue 3), suggest the way in which all three pieces were probably manufactured. Firstly, the rough form was sculpted, using the pick or double pick. Then these pieces were worked up using another tool, perhaps a tool similar to the adze which was used for working up the fragment of the leg. Finally, the fine details were made by using various types of narrow and broader chisels, as we observed in the case of the meander line on the cuirass of statue 1. The XRF analyses showed that these tools could have been made either of a copper alloy or of iron.

The results of our research on the iconic Glauberg statue and the related fragments have unveiled the technical procedures necessary to realize such a work, as well as the expertise of the Celtic sculptors. Although the number of monumental scupltures from the Celtic regions is only a small fraction compared for example to the situation in Greece, the Celtic stonemasons were able to produce sculptures which in many aspects are comparable with examples from regions with contemporary developed stonemasonry, for example in Italy or the Western Mediterranean. However, whether the skills needed to accomplish a work of art such as the Glauberg statue(s) were developed locally, based on the skills of wood-working and making quern-stones, or whether the artisans learned the techniques from the south of the Alps or from an artisan from that area, still remains unknown. This question could be answered by future research focussed on comparison with the sculpture of the mentioned regions. To answer this question, it is necessary to define the methods developed by the sculptor for working with stone. In this case, it is necessary not only to focus on the identification of the working traces of particular tools, but also on the individual phases and procedures of the shaping of the sculpture.
